# Metabolic Profile of Four Selected Cathinones in Microsome Incubations: Identification of Phase I and II Metabolites by Liquid Chromatography High Resolution Mass Spectrometry

**DOI:** 10.3389/fchem.2020.609251

**Published:** 2021-01-12

**Authors:** Beatriz T. Lopes, Maria João Caldeira, Helena Gaspar, Alexandra M. M. Antunes

**Affiliations:** ^1^Centro de Química Estrutural (CQE), Instituto Superior Técnico (IST), ULisboa, Lisboa, Portugal; ^2^BioISI – Biosystems & Integrative Sciences, Faculty of Sciences, University of Lisbon, Lisboa, Portugal; ^3^Laboratório de Polícia Científica da Polícia Judiciária (LPC/PJ), Novo edifício Sede da Polícia Judiciária, Lisboa, Portugal; ^4^MARE - Marine and Environmental Sciences Centre - Polytechnic of Leiria, Peniche, Portugal

**Keywords:** 4-CEC, 4Cl-PVP, 4-MDEC, 4-MDMC, LC-HRMS, glucuronides, Phase I, metabolites

## Abstract

Consumption of synthetic cathinones, the second largest class of new psychoactive substances (NPS) reported worldwide, represents a serious public health risk. One of the biggest challenges created by the rapid spread of NPS on the illegal drug market is the discovery of selective biomarkers for their detection in biological matrices, which is only possible through the study of their metabolic profile. The synthetic cathinones 4′-methyl-*N*,*N*-dimethylcathinone (**4-MDMC**), 4′-methyl-*N*,*N*-diethylcathinone (**4-MDEC**), 4′-chloro-α-pyrrolidinovalerophenone (**4Cl-PVP**), and 4′-chloroethylcathinone (**4-CEC**) are NPS recently seized in Europe, and, with the exception of **4-CEC**, no metabolism study was reported for these cathinones. With the ultimate goal of overcoming this gap, these cathinones were incubated *in vitro* in human and rat liver microsomes in the presence of Phase I and II (glucuronidation) co-factors, using α-pyrrolidinovalerophenone (α-PVP) as positive control. The metabolite identification was performed by liquid chromatography coupled to tandem high resolution mass spectrometry (LC-HRMS/MS). This allowed the identification of multiple Phase I and glucuronide metabolites of the selected cathinones. Additionally, a new glucuronide conjugate, derived from the recreational drug α-PVP, was herein identified for the first time. Importantly, we have demonstrated that **4-MDMC** and **4-MDEC** can act as prodrugs of the controlled substances **4-MMC** and **4-MEC**, respectively. The metabolites herein identified are expected to play an important role not only by acting as potential selective biomarkers of the intake of the synthetic cathinones selected for this study but also to understand their potential adverse effects and link these causative agents to toxicities, thereby helping in the treatment of non-fatal intoxications.

## Introduction

NPS are a range of substances, mostly synthetic, that have been emerging in the recreational drugs market with the purpose of mimicking the effects of classic drugs and circumvent legislation restrictions against illicit substances (German et al., 2014). The term NPS, according to the Directive (EU) 2017/2103 (EU, 2017) designates substances that are not covered by the Single Convention on Narcotic Drugs of 1961 neither by the Convention on Psychotropic Substances of 1971 but are likely to present health or social risks comparable to that posed by substances listed in those conventions. Despite the rapid spread of NPS over the last 10 years, the number of first detections of NPS, reported to the European Monitoring Center for Drugs and Drug Addiction (EMCDDA) through the European Union Early Warning System (EWS), has recently slowed-down, most likely due to changes in drug policies (EMCDDA and EUROPOL, 2019; EMCDDA, 2020) Nevertheless, the around 50 novel NPS that are still reported annually by EWS, together with the circulation in the market of about 400 previously reported NPS, pose serious health risks and constitute a great challenge for health and forensic institutions (EMCDDA, 2020). Importantly, until December 2019, a total of 790 NPS were monitored by EMCDDA (2017) overall, these represent more than three times the number of drugs currently controlled by international conventions.

Synthetic cathinones, the second largest group of NPS reported worldwide (UNODC, 2018; EMCDDA, 2020) are β-keto phenethylamines, structural analogs of cathinone, the major psychoactive alkaloid present in the leaves of *Catha edulis*. This plant, usually known as khat, is native from eastern Africa and southern Arabia, where it has been used, over centuries, due to its stimulant effects (Valente et al., 2014; Pieprzyca et al., 2020). In fact, synthetic cathinones are known to produce psychostimulant effects similar to methamphetamine, cocaine, or MDMA (ecstasy) by interacting with the plasma membrane transporters of the monoamine neurotransmitters, dopamine, norepinephrine, and serotonin, increasing their synaptic cleft concentration (German et al., 2014; Weinstein et al., 2017).

Synthetic cathinones were initially synthesized as an attempt to find new products with therapeutic applications due to their structural similarity with amphetamines (Kelly, 2011). Although some cathinones have been approved (FDA, 2020) and marketed as therapeutic agents, mainly as antidepressants or appetite suppressants (Arias et al., 2009; Kelly, 2011; Kang and Park, 2012; German et al., 2014; Onakpoya et al., 2016; FDA, 2020), most of them were banned or withdrawn from the market due to serious adverse effects or their potential misuse. Nonetheless, some cathinones are still in clinical use. One representative example is buproprion, which is an uncontrolled drug and is clinically used, mainly in the treatment of depression (Carroll et al., 2014; Costa et al., 2019; FDA, 2020).

The increase of acute intoxication and deaths associated with the consumption of cathinones (and other NPS) (Weinstein et al., 2017; Zaami et al., 2018) has resulted in their continuous inclusion in the International Drug Control Convention (UNODC, 2020), based on their risk assessment. Since 2015, 11 synthetic cathinones were included in the Schedule II of the Convention on Psychotropic Substances of 1971 (UNODC, 2020): mephedrone (**4-MMC**), methylenedioxypyrovalerone (**MDPV**), and methylone (in 2015); α-pyrrolidinopentiophenone (**α-PVP**) (in 2016); 4′-methyl-*N*-ethylcathinone (**4-MEC**), pentedrone and ethylone (in 2017); ephylone (in 2019); 4′-chloromethcathinone (**4-CMC**), *N*-ethylhexedrone (**NEH**), and α-pyrrolidinohexanophenone (**α-PHP**) (in 2020).

Metabolic profiling of cathinones is crucial not only to understand potential adverse effects and link causative agents to toxicities but also to identify unique biomarkers of their intake, that will allow their unequivocal identification in biological matrices. Some cathinones undergo extensive metabolism *in vivo* (Uralets et al., 2014; Ellefsen et al., 2016), which is translated into low or even negligible urinary levels of the parent cathinone. Therefore, for these cases, the detection of cathinone's metabolites in biofluids, is the only possible way of proving the consumption of these NPS, in particular several hours after its intake. However, the metabolic profiles of most of the recently reported synthetic cathinones remain unknown. In fact, the large number of new substances that appear in the market makes it difficult for the authorities to respond with the needed analytical methodologies and metabolic studies for the identification and quantification of parent NPS and their metabolites in biofluids. In this regard, *in vitro* approaches, in particular liver microsome incubations, have proved their utility for studying the metabolic profile of cathinones (Meyer et al., 2010, 2012; Pedersen et al., 2013; Helfer et al., 2015; Negreira et al., 2015; Pozo et al., 2015).

**Figure 1:**
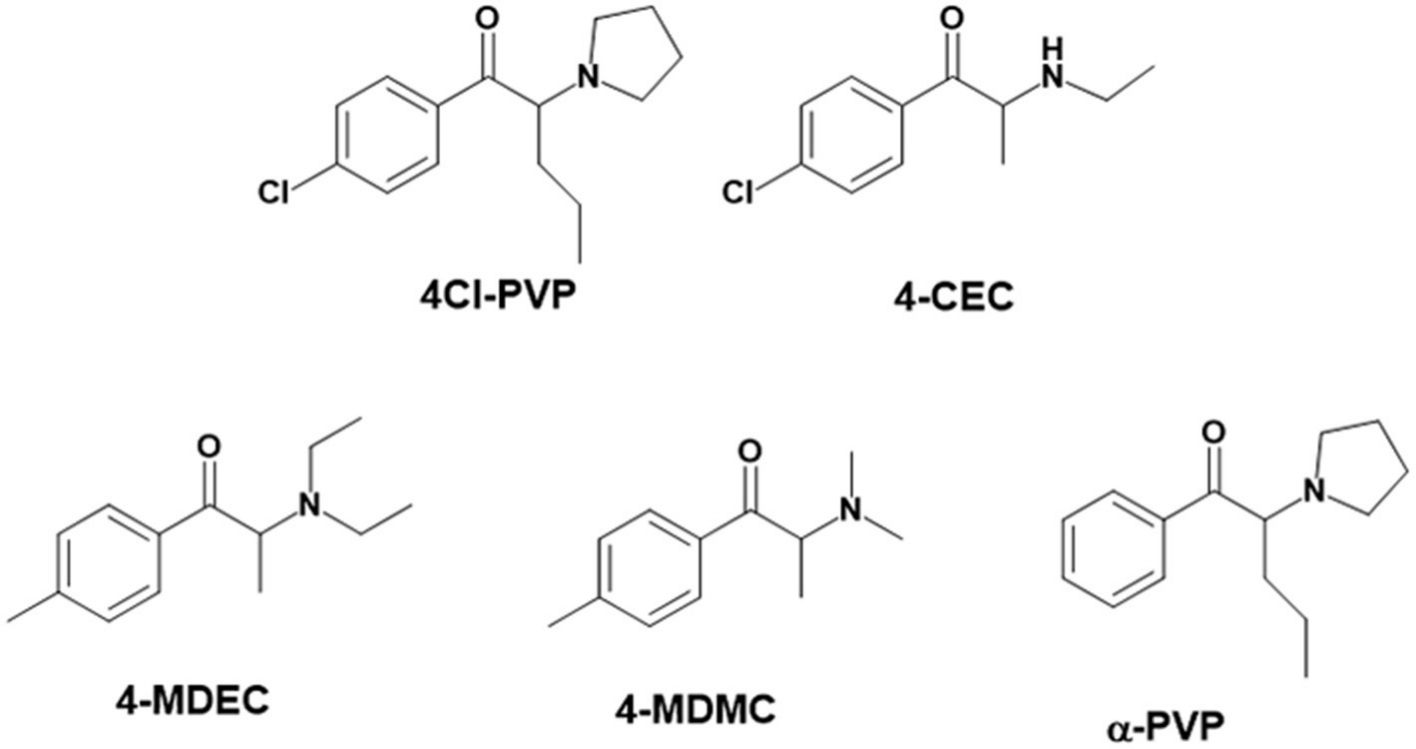
Structures of the synthetic cathinones used in the current study.

With the ultimate goal of contributing for a proactive response in tackling the NPS problem, and within the scope of a protocol established between the Forensic Science Laboratory from Portuguese Criminal Police and the Lisbon University (Instituto Superior Técnico and Faculty of Sciences), the present work is aimed at determining the metabolite profile of four selected *para*-substituted cathinones: 4′-methyl-*N,N*-dimethylcathinone (**4-MDMC**), 4′-methyl-*N,N*-diethylcathinone (**4-MDEC**), 4′-chloro- α-pyrrolidinovalerophenone (**4Cl-PVP**) and 4′-chloro-*N*-ethylcathinone (**4-CEC**) (Figure 1). The cathinone **4-MDMC** is a structural isomer of the controlled drug **4-MEC** and pentedrone. **4-MDMC** is structurally-related to amfepramone (**DMC**) and **4-MDEC** is an isomeric structure of the recently scheduled drug **NEH**. Both these cathinones were seized for the first time during 2014 (EMCDDA, 2015) **4Cl-PVP** and **4-CEC** were reported in 2015 (EMCDDA, 2016) and 2016 (EMCDDA, 2017), respectively. Importantly, **4-MDEC** was the most cytotoxic cathinone tested in HepG2 cells, in our previous work (Gaspar et al., 2018) and **4-MDMC** was one of the five most seized cathinones in EU, during 2016, while **4-CEC** was the second one with highest overall quantities of seized products (EMCDDA, 2017). All the four selected cathinones are controlled in Portugal (Decree Law 15/93 and Decree Law 54/2013) and only **4-CEC** has already been subject of metabolism studies (Fabregat-Safont et al., 2020; Wagmann et al., 2020).

## Materials and Methods

### Chemicals and Biochemicals

The analytical standards of the hydrochloride salts of the selected cathinones (purity >97%) used in this work were previously obtained and characterized within the scope of the protocol established between the Faculty of Sciences of the University of Lisbon (FCUL) and the Forensic Science Laboratory from Portuguese Criminal Police (LPC-PJ): **4-MDMC**, **4-MDEC** and **α-PVP** were synthesized at FCUL, as described by Gaspar et al. (2018), while **4Cl-PVP** and **4-CEC** were obtained from seized samples provided by LPC-PJ (Antunes et al., 2020). Rat and human liver microsomes were obtained from Thermo Fisher Scientific-Gibco. All other commercially available reagents were acquired from Sigma-Aldrich Química, S.A. and used as received.

### Incubation of Cathinones With Human and Rat Liver Microsomes

#### Generation of Phase I Metabolites

Cathinones, at a concentration of 10 μM (1 μL, 5 mM water solution), were incubated with human liver microsomes (HLM) and rat liver microsomes (RLM) (1 mg/mL), NADPH (20 mM, 1 μL), for a total incubation volume of 200 μL in 50 mM ammonium bicarbonate buffer at pH 7.4. Each incubation was run in duplicate. Control incubations were conducted in the same conditions: (1) using water as a negative control, in the absence of cathinone; (2) in the absence of the NADPH cofactor; and (3) using heat-denatured (90°C, 15 min) microsomes. Incubation of **α-PVP**, was run at the same conditions, as a positive control incubation. The mixtures were incubated at 37°C and 100 μL aliquot was collected following 2 h of incubation. Acetonitrile (100 μL) was then added to quench the reactions. Following centrifugation at 10,000 g for 15 min at room temperature, the supernatants were collected and analyzed by liquid chromatography-tandem high resolution mass spectrometry (LC-HRMS/MS).

#### Generation of Phase II Metabolites

HLM and RLM (1 mg/mL) were preincubated for 15 min, in ice, with alamethicin (4 μL, 5 mgmL^−1^) in 50 mM ammonium bicarbonate buffer at pH 7.4, for a total incubation volume of 200 μL. Following the addition of MgCl_2_ (400 mM; 1 μL) and cathinone (10 μM final concentration; 1 μL, 5 mM water solution), the resulting solution was incubated for 5 min at 37°C. NADPH (20 mM, 1 μL) and UDPGA (250 mM, 10 μL) were then added to start the Phase I and II reactions. Incubations were run in duplicate. Control incubations were conducted in the same conditions: (1) using water as a negative control, in the absence of cathinone; (2) in the absence of the NADPH and UDPGA cofactors; and (3) using heat-denatured (90°C, 15 min) microsomes. Incubation of **α-PVP**, was run at the same conditions, as a positive control incubation. The mixtures were incubated at 37°C and a 100 μL aliquot was collected following 2 h of incubation. Acetonitrile (100 μL) was then added to quench the reactions. Following centrifugation at 10,000 g for 15 min at room temperature, the supernatants were collected and analyzed by LC-HRMS/MS.

### Liquid Chromatography-Tandem High Resolution Mass Spectrometry (LC-HRMS/MS) Analyses

LC-HRMS/MS analyses were performed on a Bruker Impact II quadrupole time-of-flight mass spectrometer equipped with an ESI source (Bruker Daltoniks, Bremen, Germany). Chromatographic separations were performed on: (1) an Ultimate 3000 RSLCnano system (ThermoFisher Scientific) using a Luna C18 column (3.0 μm, 2.0 × 150 mm; Phenomenex) and an elution gradient of 0.1% formic acid in water (mobile phase A) and 0.1% formic acid in acetonitrile (mobile phase B) at a flow rate of 170 μL/min. The elution conditions were as follows: 5–50% B for 6 min; 50–100% B for 4 min; isocratic elution with 100% B for 5 min; 100–5% B for 4 min; and finally, 5% B for 9 min; or (2) an Ultimate 3000 RSLCnano system (ThermoFisher Scientific) using a HypersilGold C18 column (2.1 × 150 mm,1.9 μm particle size; ThermoFisher Scientific) at a flow rate of 200 μL/min. The elution conditions were as follows: 5% B for 2.4 min; 5–25% B for 2.1 min; 25–70% B for 4.1 min; 70–100% B for 3 min; 100% B for 3 min; 100–5% B for 2 min; and finally 5% B for 6 min. In either instance, the injection volume was 10 μL. The column and the autosampler were maintained at 40 and 8°C, respectively. The mass spectrometric parameters were set as follows: end plate offset, 500 V; capillary voltage, 4.5 kV; nebulizer, 40 psi; dry gas, 8 L/min; heater temperature, 200°C. Spectra were acquired in the positive electrospray ionization mode ESI (+). Internal calibration was performed for sodium formate cluster, with a sodium formate solution introduced to the ion source via a 20 μL loop at the beginning of each analysis using a six-port valve. Calibration was then performed using high-precision calibration mode (HPC). Acquisition was performed in the *m/z* 50–1,000 range and in a data-dependent MS/MS mode with an isolation window of 0.5, acquisition rate of 3 Hz and a fixed cycle time of 3 s. Precursor ions were selected for auto MS/MS at an absolute threshold of 153, with the active exclusion mode set at three spectra and released after 1 min, but precursor ions with intensities in the range of 5x the previous intensities were reconsidered.

### Data Processing

The acquired data were processed by DataAnalysis 4.1 software (Bruker Daltoniks). Extracted ion chromatograms (EIC), with a mass window of ± 5 ppm, were performed for searching the protonated molecule of the expected metabolites in the full scan spectra. Isotope cluster analysis was also used to identify chlorinated metabolites. All spectra corresponding to metabolites were then manually checked. The mass deviation from the accurate mass of the identified cathinone metabolites remained below 5 ppm for the precursor and below 10 ppm for product ions. The MS/MS spectra of the four selected cathiones and their identified metabolites are displayed in the Supplementary Material (Supplementary Figures 1–20).

## Results and Discussion

Taking into consideration that liver microsome incubations were already successfully used for the generation of metabolites that occur *in vivo* for other cathinones (Meyer and Maurer, 2010; Meyer et al., 2010, 2012; Strano-Rossi et al., 2010; Negreira et al., 2015), this *in vitro* model was used for the identification of the metabolite profile of the four *para*-substituted cathinones, **4-MDMC**, **4-MDEC**, **4Cl-PVP**, and **4-CEC**, selected for this study. HLM and RLM incubations were run for each of these cathinones. This decision was made based on our previous work (Godinho et al., 2018), where considerably more metabolites were identified in the incubations of the anti-HIV drug etravirine run in the rat liver S9 fraction when compared with the corresponding human liver S9 fraction incubations, due possibly to the higher enzymatic activity of the rodent fraction. Nonetheless, importantly, metabolites that were solely identified *in vitro* in the rat liver S9 incubations were also identified *in vivo* in the urine of HIV patients in etravirine therapy. Therefore, the fact that a specific metabolite is only detected in RLM does not necessarily mean that this metabolite cannot be formed *in vivo* in humans. However, in the current study, with very few exceptions, which will be conveniently mentioned, the metabolites were consistently identified in HLM and RLM incubations. This is particularly relevant since most *in vivo* toxicological studies are performed in rodent models (Prosser and Nelson, 2012).

For the identification of Phase I metabolites, incubations were run in the presence of NADPH co-factor. Glucuronidation of Phase I metabolites, and urinary excretion of the glucuronide conjugates represent the primary routes of cathinones biotransformation and elimination in humans (Ellefsen et al., 2016). Therefore, toward the identification of glucuronic acid conjugates of the selected cathinones, additional incubations were run in the presence of Phase I and II cofactors, NADPH and UDPGA, respectively, using both HLM and RLM that were preincubated with the pore-forming peptide alamethicin. This procedure was already proved to increase considerably the rates of the Phase II glucuronidation, while not effecting the Phase I capacity (Fisher et al., 2000). Since the metabolite profile of the drug **α-PVP** was already determined *in vivo* (Shima et al., 2014) and *in vitro* (Negreira et al., 2015), **α-PVP** incubations were run in parallel with the incubations of selected cathinones, as positive controls.

LC-HRMS/MS, using hybrid quadrupole time-of-flight (QTOF) analyzer, was the method of choice for establishing the metabolic profile of the selected cathinones, since it constitutes one of the most useful analytical platforms for the identification of unknown metabolites. In fact, this methodology allows the direct and simultaneous detection of both Phase I and II metabolites, providing information about accurate mass and isotopic pattern not only of intact (de)protonated molecules, but also of fragment ions, which is of particular usefulness for metabolite identification.

**Figure 2:**
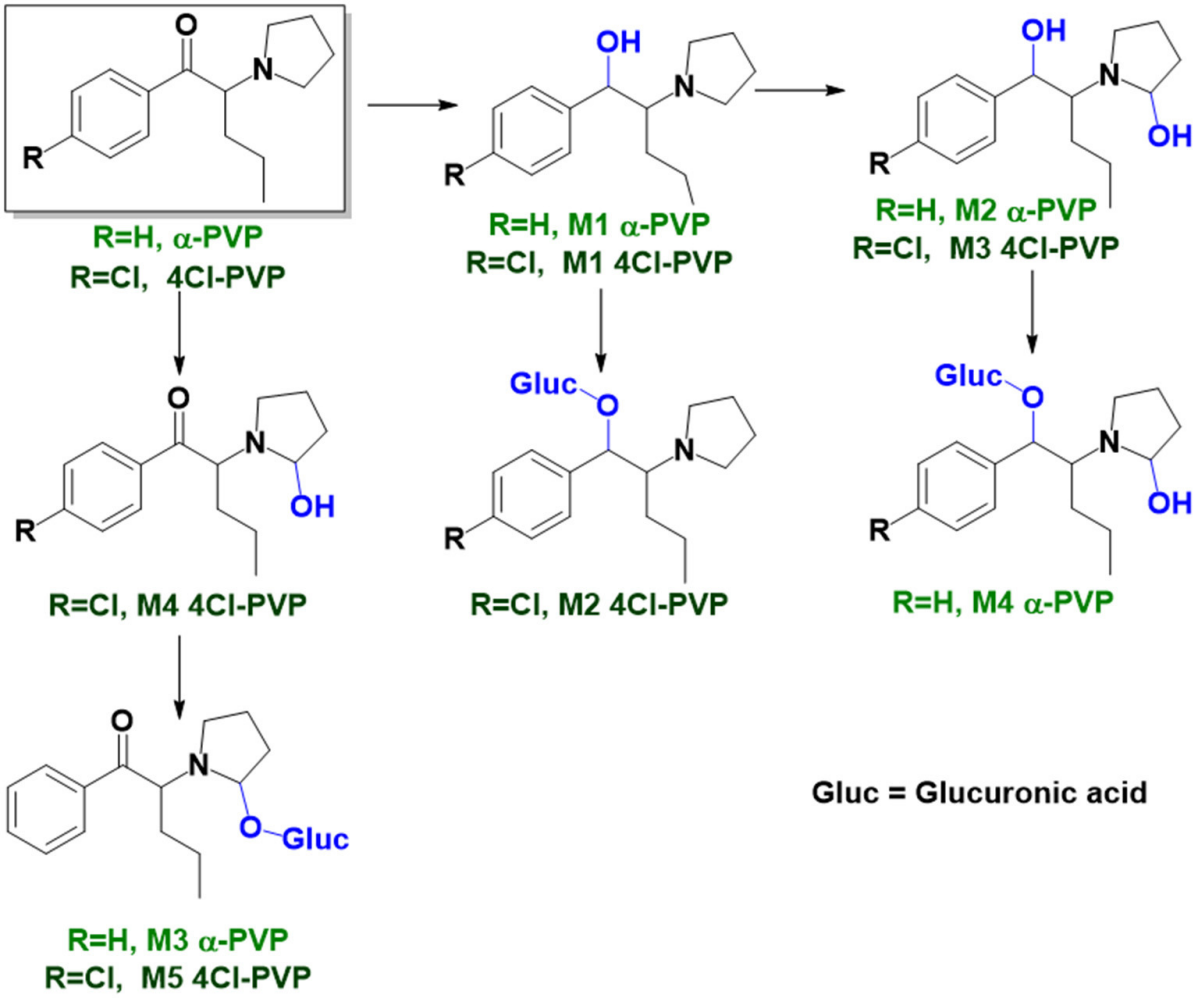
Proposed structures of the **α-PVP** and **4Cl-PVP** Phase I and II metabolites identified by LC-HRMS/MS analysis in HLM and RLM incubations.

As stated above, **α-PVP** incubations were performed in this study as positive controls. The fragmentation pattern obtained for this cathinone (Table 1, Supplementary Figure 1) is in agreement with the one reported by Negreira et al. (2015). Likewise, all Phase I **α-PVP** metabolites identified in current study (Table 1, Figure 2, Supplementary Figures 2–5) were also identified by these authors *in vitro*, including the product of **α-PVP** ketone reduction (**M1** **α-PVP**), which was the most abundant metabolite found in *in vivo* for this cathinone (Tyrkkö et al., 2013). This result attests the viability of the experimental conditions used in the current study for identifying the metabolites that are most likely to be found *in vivo*. Of note is the identification of the Phase II metabolite **M4** **α-PVP** (*m/z* 426.2118, −0.9 ppm, C_21_H_32_NO_8_) that stems from glucuronic acid conjugation of **M2** **α-PVP**, a Phase I metabolite formed upon **α-PVP** keto reduction and hydroxylation of the pyrrolidine ring (Figure 2). The base peak of the tandem mass spectrum of this Phase II metabolite, at *m/z* 250.1803 (+0.8 ppm, C_15_H_24_NO_2_) (Supplementary Figure 5), corresponds to the typical neutral loss of 176.0315 u from the protonated molecule, corresponding to the glucuronide moiety. The occurrence of glucuronidation at the hydroxyl group resulting from the reduction of the keto group of the parent cathinone is suggested based on the observation of the product ion at *m/z* 161.0953 (+5.0 ppm, C_11_H_13_O). In fact, the formation of this product ion can only be explained on basis of the loss of the hydroxylated pyrrolidine moiety from the fragment ion at *m/z* 248.1653 (+3.2 ppm, C_14_H_19_NO_2_), which is proposed to be formed via the neutral loss of 178.0465 u from the protonated molecule of **M4** **α-PVP**. Whereas, the *in vivo* occurrence of the parent Phase I metabolite was already evidenced by Tyrkkö et al. (2013) to the best of our knowledge, this constitutes the first report on the identification of the glucuronic acid conjugate of this **α-PVP** Phase I metabolite. Coherently, the product ion displayed by metabolite **M4** **α-PVP** is distinct from the one reported by Negreira et al. (2015) for its isomer, resulting from the glucuronidation of the hydroxyl group of the Phase I metabolite stemming from reduction of **α-PVP** combined with a hydroxylation in the pyrrolidine ring and a subsequent oxidation. Interestingly whereas **M2** **α-PVP** was only identified in HLM incubations, its Phase II metabolite was identified in both RLM and HLM, thereby suggestion that the parent Phase I metabolite was also formed in RLM.

**Table 1:** Structures of the **α-PVP** metabolites identified in HLM and RLM incubations by LC-HRMS/MS (ESI+) analysis.

**Compound**	**Structure**	**[M+H]^**+**^ (*m/z*)**	**Elemental composition**	**Error (ppm)**	**Production *(m/z)***	**Elemental composition**	**Error (ppm)**	**Identification**
α-PVP	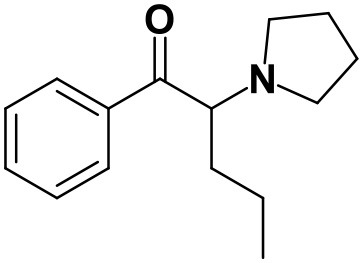	232.1685	C_15_H_22_NO	−4.7	161.0964	C_11_H_13_O	+2.5	
					126.1281	C_8_H_16_N	+3.2	
					105.0343	C_7_H_5_N	+7.6	–
					91.0541	C_7_H_7_	−1.9	
					70.0650	C_4_H_8_N	−1.4	
M1 α-PVP	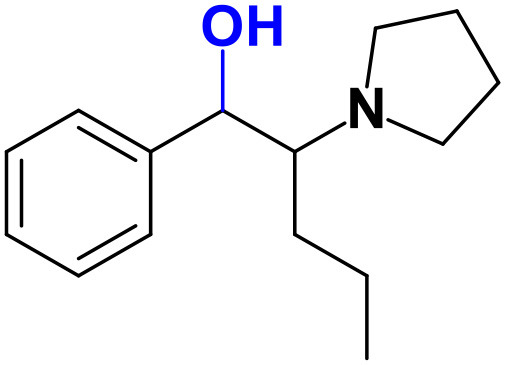	234.1849	C_15_H_24_NO	−1.3	216.1736	C_15_H_22_N	−5.1	
					173.1194	C_12_H1_5_N	−2.8	HLM
					91.0539	C_7_H_7_	−3.3	RLM
M2 α-PVP	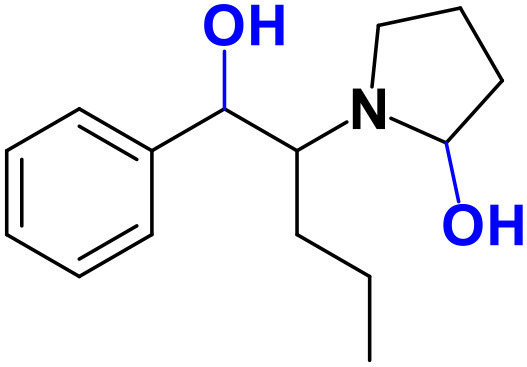	250.1809	C_15_H_24_NO_2_	+2.8	232.1706	C_15_H_22_NO	−4.3	HLM
					214.1591	C_15_H_20_N	−0.5	
M3 α-PVP	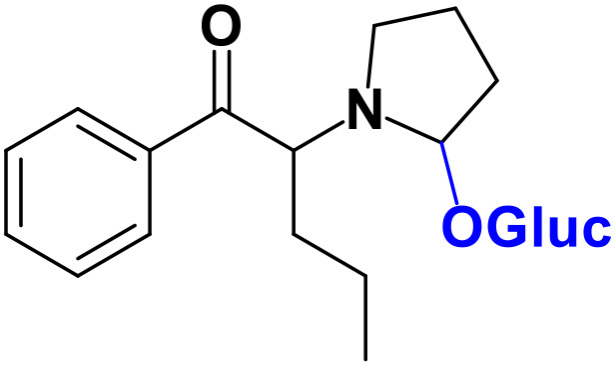	424.1972	C_21_H_30_NO_8_	+1.4	48.1645	C_15_H_22_NO_2_	0	
					230.1538	C_15_H_20_NO	−0.4	HLM
					161.0960	C_11_H_13_O	−0.6	RLM
M4 α-PVP	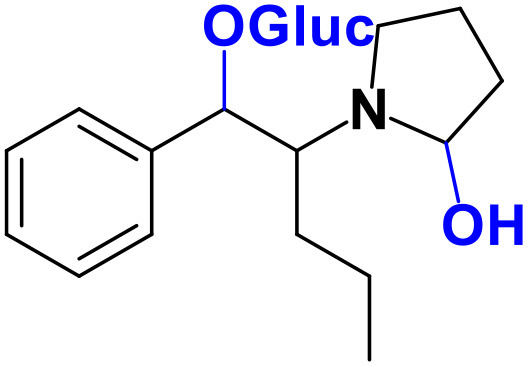	426.2118	C_21_H_32_NO_8_	−0.9	250.1803	C_15_H_24_NO_2_	+0.8	
					232.1699	C_15_H_22_NO	+1.3	HLM
					248.1653	C_14_H_19_NO_2_	+3.2	RLM
					161.0953	C_11_H_13_O	+5.0	

*Also shown the corresponding experimental m/z values and proposed elemental composition for the protonated molecule and characteristic fragment ions (presented with the associated error in ppm)*.

### 4′-Chloro-α-pyrrolidinovalerophenone (4Cl-PVP)

**4Cl-PVP** is a chlorinated derivative of **α-PVP** and, as expected, these two cathinones present similar behaviors when analyzed by LC-HRMS/MS. Presenting the protonated molecule at *m/z* 266.1311 (+1.9 ppm, C_15_H_21_ClNO), with the chlorine isotopic pattern, the product ion of this cathinone displays at least three fragmentation pathways similar to the ones observed for **α-PVP** (Supplementary Figure 1 vs. Supplementary Figure 6): (1) at *m/z* 195.0568 (−1.5 ppm, C_11_H_13_ClO) which is formed upon loss of the pyrrolidine moiety from the parent protonated molecule; (2) at *m/z* at 126.1304 (−4.7 ppm, C_8_H_16_N), which corresponds to the loss pyrrolidine and alkyl moieties from the protonated molecule; and (3) at *m/z* 138.9957 (+8.6 ppm, C_7_H_4_ClO) corresponding to the oxonium ion. Two additional product ions are observed at *m/z* 223.0753 (2.2 ppm, C_12_H_14_ClNO), which corresponds to the loss of propyl radical from the protonated molecule, and *m/z* 153.0102 (0 ppm, C_6_H_6_ClO), stemming from the loss of the pyrrolidine and propyl moieties.

The recognition of the chlorine isotopic cluster was key for the identification of **4Cl-PVP** metabolites. Two Phase I and two Phase II metabolites were consistently identified in the RLM and HLM incubations of this cathinone and a third Phase I metabolite, **M3 4Cl-PVP**, was only identified in HLM incubations (Table 2, Figure 2, Supplementary Figures 7–11).

**Table 2:** Structures of the **4Cl-PVP** metabolites identified in HLM and RLM incubations by LC-HRMS/MS (ESI+) analysis.

**Compound**	**Structure**	**[M+H]^**+**^(*m/z*)**	**Elemental composition**	**Error (ppm)**	**Productions *(m/z*)**	**Elemental composition**	**Error (ppm)**	**Identification**
4Cl-PVP	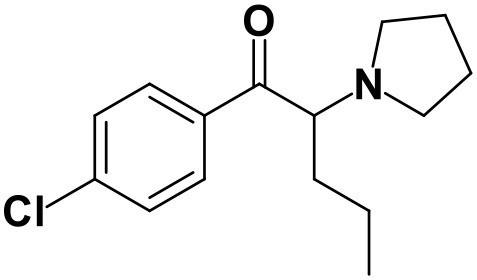	266.1311	C_15_H_21_ClNO	+1.9	223.0753	C_12_H_14_ClNO	+2.2	
					195.0568	C_11_H_13_ClO	−1.5	
					153.0102	C_8_H_6_ClO	0	
					138.9957	C_7_H_4_ClO	+8.6	
					126.1304	C_8_H_16_N	−4.7	
M1 4Cl-PVP	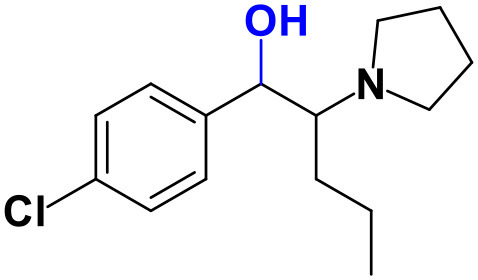	268.1463	C_15_H_23_ClNO	0	250.1359	C_15_H_21_ClN	+0.8	HLM
					207.0806	C_12_H_14_ClN	−1.4	RLM
M2 4Cl-PVP	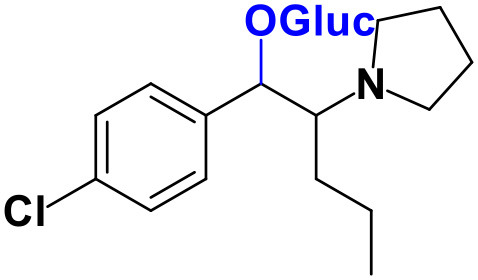	444.1786	C_21_H_31_ClNO_7_	+0.5	268.1469	C_15_H_23_ClNO	+2.2	
					250.1371	C_15_H_20_ClN	+5.6	HLM
					207.0808	C_12_H_14_ClN	−0.5	RLM
M3 4Cl-PVP	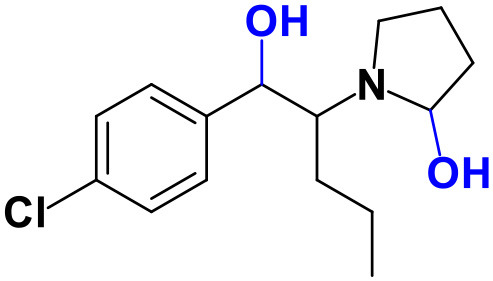	284.1413	C_15_H_23_ClNO_2_	+0.4	266.1302	C_15_H_21_ClNO	−1.5	
					248.1201	C_15_H_19_ClN	0	HLM
					206.0738	C_12_H_13_ClN	+0.5	
M4 4Cl-PVP	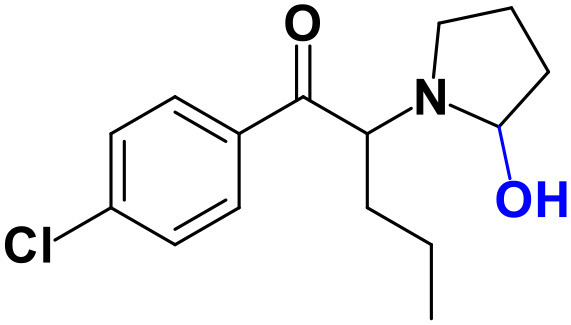	282.1256	C_15_H_21_ClNO_2_	+0.4	264.1155	C_15_H_19_ClNO	+1.9	
					142.1233	C_8_H_16_NO	+4.9	HLM
					153.0109	C_8_H_6_ClO	+4.6	RLM
M5 4Cl-PVP	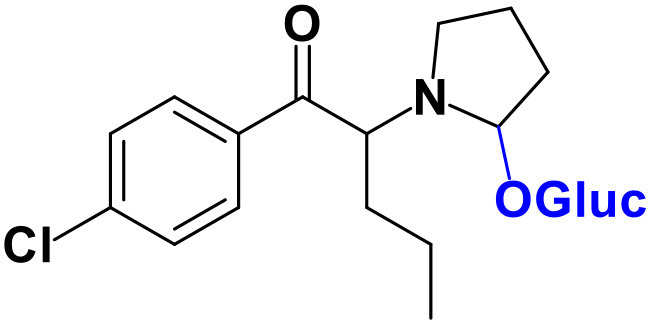	458.1580	C_21_H_29_ClNO_8_	+0.9	282.1261	C_15_H_21_ClNO_2_	+2.1	
					264.1154	C_15_H_19_ClNO	+1.5	HLM
					195.0568	C_11_H_12_ClO	−1.5	RLM

*Also shown the corresponding experimental m/z values and proposed elemental composition for the protonated molecule and characteristic fragment ions (presented with the associated error in ppm)*.

The Phase I metabolite, **M1 4Cl-PVP**, exhibits the protonated molecule at *m/z* 268.1463 (0 ppm, C_15_H_23_ClNO), compatible with the reduction of the carbonyl group of **4Cl-PVP**. This constitutes a metabolic transformation very frequent for cathinones (Zaitsu et al., 2014) and is also reported to occur for **α-PVP**, *in vivo* (Shima et al., 2014). The tandem mass spectrum of this metabolite exhibits two main fragments (Supplementary Figure 7): (1) at *m/z* 250.1359 (+0.8 ppm, C_15_H_21_ClN) that stems from the loss of water from the protonated molecule; and (2) at *m/z* 207.0806 (−1.4 ppm, C_12_H_14_ClN), which results from the subsequent loss of the propyl radical from the previous fragment ion. This fragmentation pattern is consistent with the one described for the analog **α-PVP** metabolite (Shima et al., 2014; Zaitsu et al., 2014). As stated above, ketone reduction is the major metabolic pathway reported for other α-pyrrolidinophenones in humans, thereby supporting the potential *in vivo* relevance of this metabolite for **4Cl-PVP**. The glucuronide conjugate of **M1 4Cl-PVP** was also identified. In fact, the neutral loss of 176.0317 u from the protonated molecule of **M2 4Cl-PVP** (*m/z* 444.1786, +0.5 ppm, C_21_H_31_ClNO_7_), observed in the tandem mass spectrum (Supplementary Figure 8), is indicative of glucuronidation. The observation of two fragment ions at *m/z* 250.1371 (+5.6 ppm, C_15_H_20_ClN) and 207.0808 (−0.5 ppm, C_12_H_14_ClN), which are also observed in the tandem mass spectrum of **4Cl-PVP M1**, further substantiates the assigned structure of **M2 4Cl-PVP**. The fact that a structurally similar glucuronide derived from **α-PVP** was identified as a urinary metabolite in humans, suggests that this might also be a metabolically relevant pathway for **4Cl-PVP** *in vivo* (Shima et al., 2014).

**M3 4Cl-PVP** is one additional Phase I metabolite that was identified in **4Cl-PVP** HLM incubations and results from the hydroxylation of **M1 4Cl-PVP**. Exhibiting the protonated molecule at *m/z* 284.1413 (+0.4 ppm, C_15_H_23_ClNO_2_), **M3 4Cl-PVP** displays diagnostic product ions (Supplementary Figure 9), corresponding to two consecutive losses of water: at *m/z* 266.1302 (−1.5 ppm, C_15_H_21_ClNO) and 248.1201 (0 ppm, C_15_H_19_ClN). The subsequent loss of the alkyl chain is also observed at *m/z* 206.0738 (+0.5 ppm, C_12_H_13_ClN). Whereas the product ion obtained does not allow the identification of the exact location of the hydroxyl group, the 2′ position of the pyrrolidine is suggested, since it is a frequent hydroxylation site for other α-pyrrolidinophenones, including **α-PVP** (Tyrkkö et al., 2013; Negreira et al., 2015). A similar metabolic transformation was also reported for **PV8** (α-pyrrolidinoheptaphenone) (Swortwood et al., 2016), a cathinone analog closely resembling **α-PVP**.

The mass increase of 15.994 u from **4Cl-PVP** observed for the protonated molecule of **M4 4Cl-PVP**, at *m/z* 282.1256 (−0.4 ppm, C_15_H_21_ClNO_2_), is indicative of direct hydroxylation of the parent cathinone. The observation of the product ion at *m/z* 142.1233 (+4.9 ppm, C_8_H_16_NO) (Supplementary Figure 10), suggests that this metabolite stems from the hydroxylation of the pyrodilidine or alkyl moieties. Coherently, these hydroxylation locations are also reported for **α-PVP** *in vivo* (Tyrkkö et al., 2013). Nonetheless, the consistent identification of the glucuronide **M5 4Cl-PVP** in HLM and RLM incubations suggests that the most probable location of hydroxylation in **M4 4Cl-PVP** is the pyrrolidine ring. In fact, the tandem mass spectrum of **M5 4Cl-PVP** protonated molecule, at *m/z* 458.1580 (+ 0.9 ppm, C_21_H_29_ClNO_8_), exhibits a product ion at *m/z* 195.0568 (−1.5 ppm, C_11_H_12_ClO) (Supplementary Figure 11), which results from the loss of hydroxylated and glucuronidated pyrrolidine moiety from the protonated molecule, thereby evidencing that this Phase II metabolite stems from the glucuronidation of **M4 4Cl-PVP**, bearing the hydroxyl group in the pyrrolidine moiety (Figure 2). Of note is the fact that this metabolic pathway is also observed for **α-PVP**.

### 4′-Methyl- *N,N*-dimethylcathinone (4-MDMC)

The protonated molecule of **4-MDMC** is observed at *m/z* 192.1374 (−4.7 ppm, C_12_H_18_NO) (Table 3) and its tandem mass spectrum presents a base peak at *m/z* 119.0877 (+0.2 ppm, C_9_H_11_) (Supplementary Figure 12). This fragment ion was already reported for mephedrone (**4-MMC**), when analyzed by LC-HRMS (Pozo et al., 2015), and may be explained on basis of a neutral loss containing the nitrogen after dissociation of the C-N bond from the protonated molecule, followed by CO loss. The possibility of subsequently undergoing rearrangement, with the formation of a seven-membered ring, may explain the conferred stability of this fragment (Zuba, 2012; Pedersen et al., 2013).

**Table 3:** Structures for **4-MDMC** and its Phase II metabolite identified in HLM and RLM incubations by LC-HRMS/MS (ESI+) analysis.

**Compound**	**Structure**	**[M+H]^**+**^ (*m/z*)**	**Elemental composition**	**Error (ppm)**	**Product ions *(m/z*)**	**Elemental composition**	**Error (ppm)**	**identification**
4-MDMC	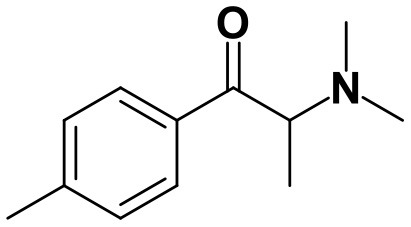	192.1374	C_12_H_18_NO	−4.7	119.0877	C_9_H_11_	+0.2	—
M1 4-MDMC	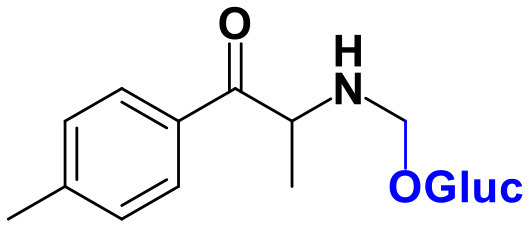	370.1488	C_17_H_24_NO_8_	−2.1	194.1167 178.1229 160.1121	C_11_H_16_NO_2_ C_11_H_16_NO C_11_H_14_N	– 4.6 +1.7 0	RLM

*Also shown the corresponding experimental m/z values and proposed elemental composition for the protonated molecule and characteristic fragment ions (presented with the associated error in ppm)*.

**Figure 3:**
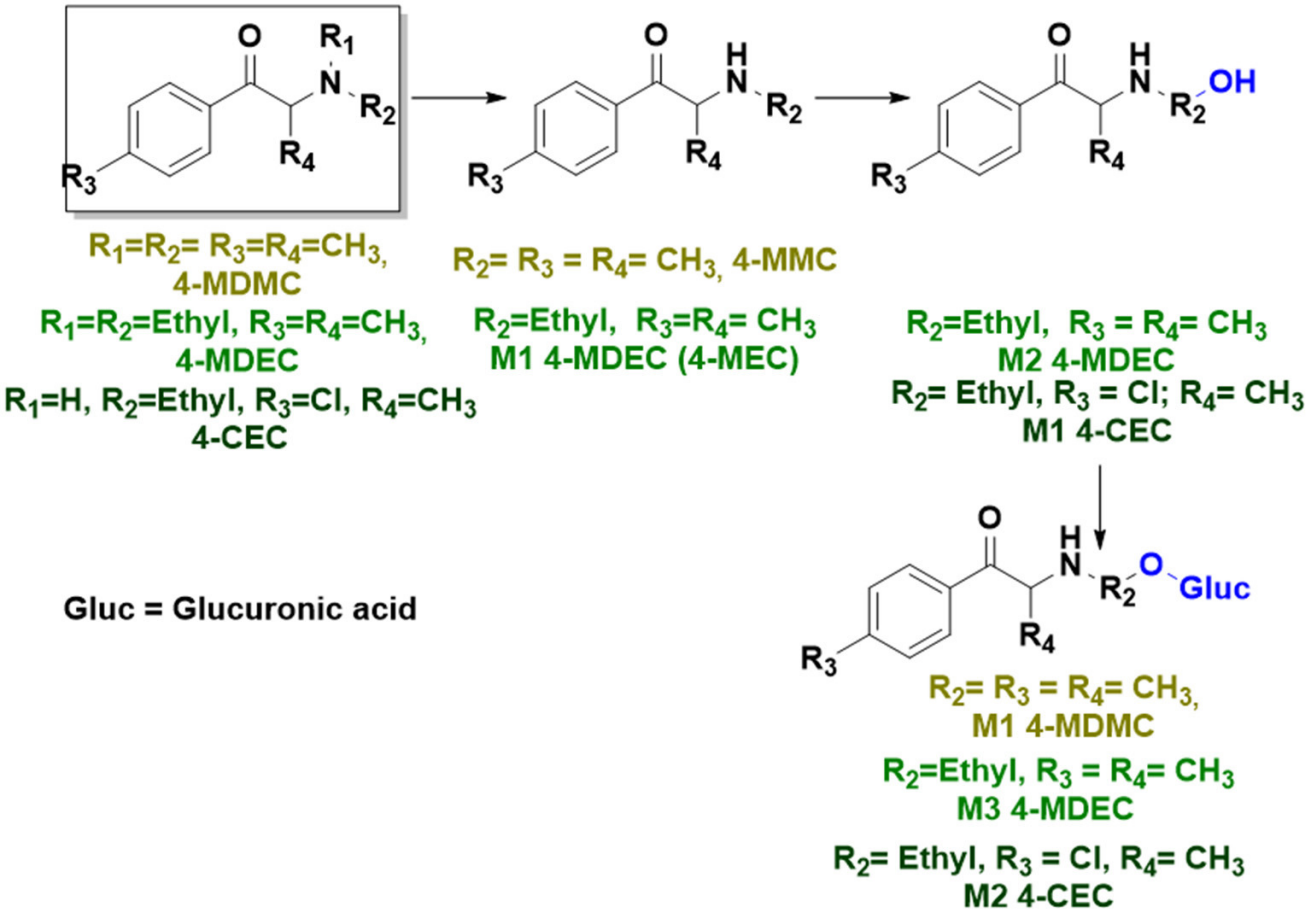
Proposed structures for the metabolites identified by LC-HRMS/MS in RLM and HLM incubations of **4-MDMC**, **4-MDEC**, and **4-CEC**.

Whereas, no Phase I metabolites were identified in **4-MDMC** incubations run in the presence of Phase I cofactors, one signal at *m/z* 370.1488 (−2.1 ppm, C_17_H_24_NO_8_), compatible with the glucuronide conjugate **M1 4-MDMC** (Table 3, Figure 3), was identified following LC-HRMS analysis of alamethicin-induced RLM incubations run in the presence of Phase I and II cofactors. **M1 4-MDMC** is compatible with the glucuronidation of a **4-MDMC** Phase I metabolite that stems from two consecutive metabolic transformations (Figure 3): **4-MDMC** demethylation, yielding mephedrone (**4-MMC**), which is subsequently hydroxylated in a methyl group. In fact, the tandem mass spectrum of **M1 4-MDMC** metabolite displays a very similar product ion profile to the one reported by Pozo et al. (2015) for a **4-MMC** Phase II metabolite, which was identified in the urine of two human volunteers that ingested 200 mg **4-MMC** orally (Pozo et al., 2015). However, whereas these authors suggest that C3 is the most probable hydroxylation site, the hydroxyl group is more likely to be at the *N*-methyl substituent. In fact, *N*-alkyl groups are considered hot spots for metabolic hydroxylation, which constitutes the first step of *N*-dealkylation that is a common metabolic pathway for substituted amines (Trager, 2007). Coherently, similarly to what was reported for this glucuronide metabolite of **4-MMC**, the tandem mass spectrum of the glucuronide **M1 4-MDMC** exhibits a base peak at *m/z* 194.1167 (−4.6 ppm, C_11_H_16_NO_2_) (Supplementary Figure 13) that results from the neutral loss of 176.0321 u from the protonated molecule, corresponding to typical the glucuronide loss. Two other minor product ions are observed at *m/z* 178.1229 (+1.7 ppm, C_11_H_16_NO) and 160.1121 (0 ppm, C_11_H_14_N), which can be explained by a mechanism similar to the one proposed by Pozo et al. (2015) (Supplementary Figure 13) for the glucuronide conjugate of the hydroxylated metabolite of **4-MMC**. Regardless of the exact location of hydroxylation of this phase I metabolite, our results suggest that **4-MDMC** may act as a prodrug of **4-MMC**. This constitutes an issue of concern taking into consideration the number of **4-MMC**-related fatalities (Busardò et al., 2015). In fact, with a very narrow safety window, **4-MMC** dosages that fall within the recreational use limits are suggested to be potentially fatal, in particular when combined with other drugs (Busardò et al., 2015). Aditionally, the fact that no **4-MDMC** was identified in the incubation where **M1 4-MDMC** was identified, suggests that this might constitute an important metabolic pathway for **4-MDMC**.

### 4′-Methyl-*N,N*-diethylcathinone (4-MDEC)

The protonated molecule of **4-MDEC** was observed at *m/z* 220.1697 (+0.5 ppm, C_14_H_22_NO) (Table 4) and whereas the fragment ion stemming from the loss of a first *N*-ethyl group is not observed in the tandem mass spectrum (Supplementary Figure 14), this constitutes the first loss undergone by this cathinone, at the experimental conditions used. In fact, the fragment ion at *m/z* 174.1270 (−4.0 ppm, C_12_H_16_N) is formed water by loss from this first product ion, a fragmentation pathway which is characteristic of other cathinones (Namera et al., 2015). The subsequent loss of CH_3_ radical explains the formation of the fragment ion at *m/z* 159.1044 (+0.6 ppm, C_10_H_12_N). Whereas the formation of radical ions is not a frequent mechanism when using ESI (Thurman et al., 2007), it seems to be particularly frequent on the LC-HRMS analysis of cathinones. In fact, this kind of fragmentation was already reported for **4-MMC** and was consistently observed in the fragmentation pattern of the glucuronide **M1 4-MDMC** (see above) (Namera et al., 2015). Likewise, the in-source rearrangement to a indole ring that was already used to explain **4-MMC** fragmentation (Pozo et al., 2015), followed by loss of the ethyl radical, can also be evoked to explain the formation of the product ion at 144.0813 (−3.5 ppm, C_10_H_10_N) observed in the **4-MDEC** tandem mass spectrum.

**Table 4:** Structures of the **4-MDEC** metabolites identified in HLM and RLM incubations by LC-HRMS/MS (ESI+) analysis.

**Compound**	**Structure**	**[M+H]^**+**^(m*/z*)**	**Elemental composition**	**Error (ppm)**	**Product ions *(m/z*)**	**Elemental composition**	**Error (ppm)**	**Identification**
4-MDEC	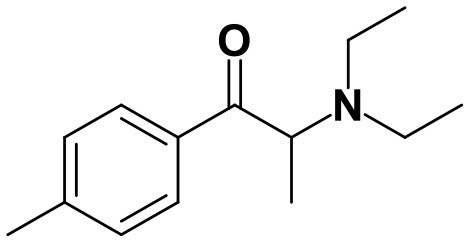	220.1697	C_14_H_22_NO	+0.5	174.1270 159.1044 144.0813	C_12_H_16_N C_10_H_12_N C_10_H_10_N	4.0 +0.6 −3.5	—
M1 4-MDEC	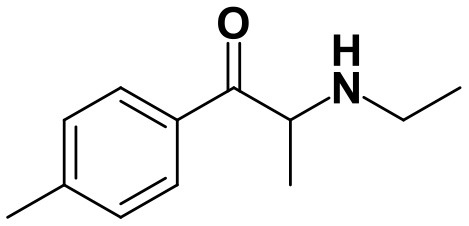	192.1378	C_12_H_18_NO	−2.6	144.0812	C_10_H_10_N	+2.8	HLM RLM
M2 4-MDEC	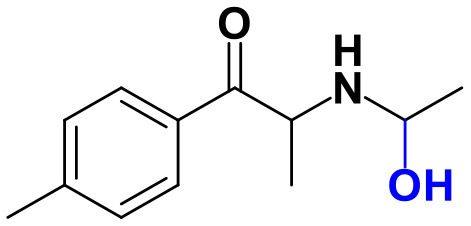	208.1329	C_12_H_18_NO_2_	+1.4	172.1128 144.0813	C_12_H_14_N C_10_H_10_N	+4.1 +3.5	HLM RLM
M3 4-MDEC	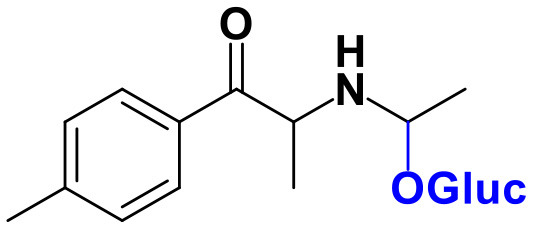	384.1637	C_18_H_26_NO_8_	+4.1	208.1337 172.1124	C_12_H_18_NO_2_ C_12_H_14_N	+2.4 +1.7	RLM

*Also shown the corresponding experimental m/z values and proposed elemental composition for the protonated molecule and characteristic fragment ions (presented with the associated error in ppm)*.

Two Phase I metabolites were consistently identified in **4-MDEC** HLM and RLM incubations (Table 4, Figure 3, Supplementary Figures 15, 16). The protonated molecule of **M1 4-MDEC** was observed at *m/z* 192.1378 (−2.6 ppm, C_12_H_18_NO), which is compatible with the protonated molecule **4-MEC**. This product of deethylation of **4-MDEC** is also a controlled drug that have been linked with several overdose cases (Gil et al., 2013; Rojek et al., 2014). However, contrary to what was reported by Helfer et al. (2015), the initial fragmentation step of **4-MEC** was not the loss of water. In fact, at the experimental conditions used, the base peak of the tandem mass spectrum of **M1 4-MDEC** (**4-MEC**) was observed at *m/z* 144.0812 (−2.8 ppm, C_12_H_18_NO) which stems from the loss of 48.0566 u from the protonated molecule. In accordance with what was already discussed, the formation of a more stable indole system can be postulated for the formation of this product ion (Supplementary Figure 15). This suggests that **4-MDEC** is metabolically converted into the controlled psychotic substance **4-MEC**.

One additional Phase I metabolite, **M2 4-MDEC**, was observed at *m/z* 208.1329 (−1.4 ppm, C_12_H_18_NO_2_). The mass increment of 15.9944 u from **M1 4-MDEC** (**4-MEC**), suggests that **M2 4-MDEC** was formed upon hydroxylation of **4-MEC**. A similar metabolite was identified by Helfer et al. (2015) upon GC-MS analysis of **4-MEC** incubations in HLM. Whereas the location for **4-MEC** hydroxylation is proposed to be the methyl substituent of the aromatic moiety (Helfer et al., 2015), no LC-HRMS data is provided for this metabolite, thereby precluding any comparison with our data. Nonetheless, the fact that the base peak, at *m/z* 144.0823 (+3.5 ppm, C_10_H_10_N), of the **M2 4-MDEC** tandem mass spectrum is the same observed for **M1 4-MDEC** (Supplementary Figure 16 vs. Supplementary Figure 15), suggests that this metabolite results from hydroxylation at the *N*-ethyl substituent. **M3 4-MDEC** is one additional Phase II metabolite that was identified in RLM incubations (Table 4, Figure 3). The protonated molecule of this glucuronide is displayed at *m/z* 384.1637 (+4.1 ppm, C_18_H_26_NO_8_) and the tandem mass spectrum of this ion (Supplementary Figure 17) shows the characteristic loss of the glucuronide moiety (176.0300 u) at *m/z* 208.1337 (+2.4 ppm, C_12_H_18_NO_2_). One additional fragment ion is observed at *m/z* 172.1124 (+1.7 ppm, C_12_H_14_N), stemming from two consecutive losses of water. Taking into consideration that the same fragment ion is also observed in the tandem spectrum of **M2 4-MDEC**, **M3 4-MDEC** is suggested to be the glucuronic acid conjugate of this Phase I metabolite. The fact that no parent cathinone was detected in the incubations where this Phase II metabolite was identified, suggests that this might constitute a relevant metabolic pathway of **4-MDEC**.

### 4′-Chloro-*N*-ethylcathinone (4-CEC)

The protonated molecule of **4-CEC** is observed at *m/z* 212.0837 (0 ppm, C_11_H_15_ClNO), exhibiting the chlorine isotope cluster. This isotopic pattern is also observed in the first product ion at *m/z* 164.0252 (−5.5 ppm, C_9_H_7_ClN), corresponding to the rearrangement product to an indole ring, following the loss of *N*-substituent and water from the protonated molecule (Supplementary Figure 18) (Nóbrega and Dinis-Oliveira, 2018). The fragment ion at *m/z* 159.1036 (−3.8 ppm, C_11_H_13_N) is formed from the consecutive loss of Cl radical and water from the protonated molecule. The subsequent loss of CH_3_ radical, explains the formation of the base peak, at *m/z* 144.0813 (+3.5 ppm, C_10_H_10_N).

A first attempt to investigate the metabolic profile of **4-CEC** was performed in human hepatocytes (Fabregat-Safont et al., 2020). However, no metabolites were identified for this cathinone in this *in vitro* system. Nonetheless, Wagmann et al. (2020) reported the metabolic profile of this cathinone in liver microsome and S9 fraction incubations, thereby enabling the identification of several Phase I and Phase II metabolites. In the current work, the recognition of the chlorine isotopic cluster, and the use of the Data Analysis isotope Cluster analysis tool, were key for the identification of additional Phase I and Phase II metabolites of **4-CEC** that were identified in RLM incubations (Table 5, Figure 3, Supplementary Figures 19, 20). **M1 4-CEC** metabolite, presents a mass increase of 15.9942 u from **4-CEC**, exhibiting the protonated molecule at *m/z* 228.0779 (−3.1 ppm, C_11_H_15_ClNO_2_) (Supplementary Figure 19). This mass increase is compatible with a hydroxylation. The observation of the base fragment ion at *m/z* 138.9947 (+1.4 ppm, C_7_H_4_ClO), corresponding to the oxonium ion, suggests that hydroxylation occurred in one of the alkyl carbons of **4-CEC**. Therefore, taking into consideration the arguments presented for the hydroxylation location of **M2 4-MDMC**, the *N*-ethyl group of **M1 4-CEC** was suggested as the most likely hydroxyl location. The glucuronide conjugate of this Phase II metabolite, **M2 4-CEC**, was also identified. Coherently, the protonated molecule of **M2 4-CEC**, *m/z* 404.1104 (+0.7 ppm, C_17_H_23_ClNO_8_), displays a product ion at *m/z* 228.0780 (+2.6 ppm, C_11_H_15_ClNO_2_), corresponding to the loss of the glucuronic acid moiety from the protonated molecule (Supplementary Figure 20). Importantly, none of these metabolites were reported by Wagmann et al. (2020). As stated above, taking into consideration the group experience (Godinho et al., 2018), the fact that these metabolites were solely identified in RLM does not necessarily imply that they cannot be also formed in humans, *in vivo*.

**Table 5:** Structures of the 4-CEC metabolites identified in RLM incubations by LC-HRMS/MS (ESI+) analysis.

**Compound**	**Structure**	**[M+H]^**+**^(*m/z*)**	**Elemental composition**	**Error (ppm)**	**Fragmentions (*m/z*)**	**Elemental composition**	**Error (ppm)**	**Identification**
4-CEC	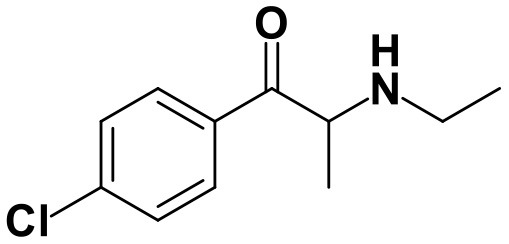	212.0837	C_11_H_15_ClNO	0	164.0252 159.1036 144.0813	C_9_H_7_ClN C_11_H_13_N C_10_H_10_N	+5.5 −3.8 +3.5	–
M1 4-CEC	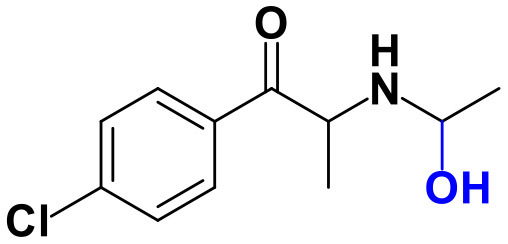	228.0779	C_11_H_15_ClNO_2_	−3.1	138.9947	C_7_H_4_ClO	+1.4	RLM
M2 4-CEC	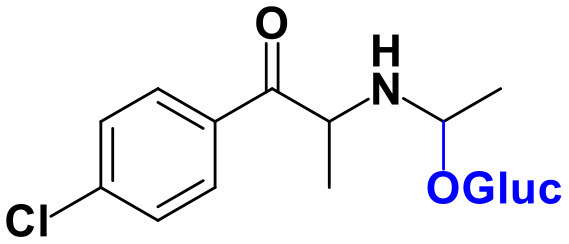	404.1104	C_17_H_23_ClNO_8_	+0.7	228.0780	C_11_H_15_ClNO_2_	−2.6	RLM

*Also shown the corresponding experimental m/z values and proposed elemental composition for the protonated molecule and characteristic fragment ions (presented with the associated error in ppm)*.

## Conclusion

Using LC-HRMS/MS analysis of HLM and RLM incubations multiple Phase I and glucoronide metabolites were identified for the selected synthetic cathinones: –**4Cl-PVP, 4-CEC, 4-MDEC, and 4-MDMC**. Specifically: (1) four Phase I and two Phase II metabolites were identified for the chlorinated derivative of α-PVP, **4Cl-PVP**; (2) one Phase I and one glucuronide conjugate were identified in RLM for **4-CEC**, which is among the most seized cathinones in 2017; (3) two Phase I and one Phase II metabolites were identified for **4-MDEC**; and (4) one Phase II metabolite was identified for **4-MDMC**. Additionally, a new Phase II conjugate, derived from the recreational drug **α-PVP**, was herein identified for the first time. Of note is the fact that this metabolic pathway, consisting on hydroxylation of the alkyl nitrogen substituent, followed by glucuronidation, was consistently observed in all cathinones studied in the current work. The reduction of the carbonyl group constituted one additional metabolic pathway for pyrrolidinic cathinones. Whereas, the metabolic profiles of these cathinones were obtained *in vitro*, the identification of metabolites stemming from metabolic pathways frequently identified *in vivo* for this class of compounds, suggests the likelihood of a similar metabolic profile being observed *in vivo*. Also noteworthy is the fact that our results suggest that **4-MDMC** and **4-MDEC** might act as prodrugs of the controlled substances **4-MMC** and **4-MEC**, respectively. These findings constitute an important contribute not only for the unequivocal proof of the intake of these recently reported synthetic cathinones but also to understand their potential adverse effects and link causative agents to toxicities.

## Data Availability Statement

The original contributions presented in the study are included in the article/Supplementary Material, further inquiries can be directed to the corresponding author/s.

## Author Contributions

HG and AA planned the work, interpreted the data, and wrote the article. BL performed the experimental and data processing steps. MC critically revised the manuscript. All authors approved the final version of the manuscript.

## Conflict of Interest

The authors declare that the research was conducted in the absence of any commercial or financial relationships that could be construed as a potential conflict of interest.

## Abbreviations

4-CEC: 4′-chloro-*N*-ethylcathinone
4Cl-PVP: 4′-chloro-α-pyrrolidinovalerophenone
4-CMC: 4′-chloro-*N*-methylcathinone
ESI+: electrospray ionization in the positive mode
HLM: human liver microsomes
LC- HRMS/MS: Liquid chromatography coupled to tandem high-resolution mass spectrometry
4-MDEC: 4-methyl-*N,N*-diethylcathinone
4-MDMC: 4-methyl-*N,N*-dimethylcathinone
4-MEC: 4′-methyl-*N*-ethylcathinone
MS: mass spectrometry
4-MMC: 4′-methyl-*N*-methylcathinone
MS/MS: tandem mass spectrometry
*m/z*: mass-to-charge ratio
NADPH: 5'-phosphate adenosine 2',5'-bisphosphate in its reduced form
NEH: *N*-ethylhexedrone
α-PHP: α-pyrrolidinohexanophenone
α-PVP: α-pyrrolidinopentiophenone
RLM: rat liver microsomes
QTOF: hybrid quadrupole time-of-flight
UDPGA: uridine diphosphate glucuronic acid.
