# Supplementary material for: Metabolic Profile of Four Selected Cathinones in Microsome Incubations: Identification of Phase I and II Metabolites by Liquid Chromatography High Resolution Mass Spectrometry
Source: Front Chem. 2021 Jan 12;8:609251. doi: 10.3389/fchem.2020.609251 (PMC7835677; doi:10.3389/fchem.2020.609251)
Supplement: Supplementary file 1 [file Data_Sheet_1.PDF]

Suplementar Material

Metabolic Profile of Four Selected Cathinones in microsome incubations: Identification of Phase I and II metabolites by Liquid Chromatography High Resolution Mass Spectrometry

Beatriz T. Lopes,[1,2] Maria João Caldeira,[3] Helena Gaspar,[2,4*] Alexandra M. M. Antunes[1*]

[1] Centro de Química Estrutural (CQE), Instituto Superior Técnico (IST), ULisboa, Avenida Rovisco Pais, 1049-001 Lisboa, Portugal
[2] BioISI – Biosystems & Integrative Sciences Institute, Faculty of Sciences, University of Lisbon, Campo Grande, C8, 1749-016 Lisboa, Portugal
[3] Laboratório de Polícia Científica da Polícia Judiciária (LPC/PJ), Novo edifício Sede da Polícia Judiciária, Rua Gomes Freire 1169-007 Lisboa, Portugal
[4] MARE - Marine and Environmental Sciences Centre - Polytechnic of Leiria, Peniche, Portugal, Avenida do Porto de Pesca, 2520-630 Peniche, Portugal

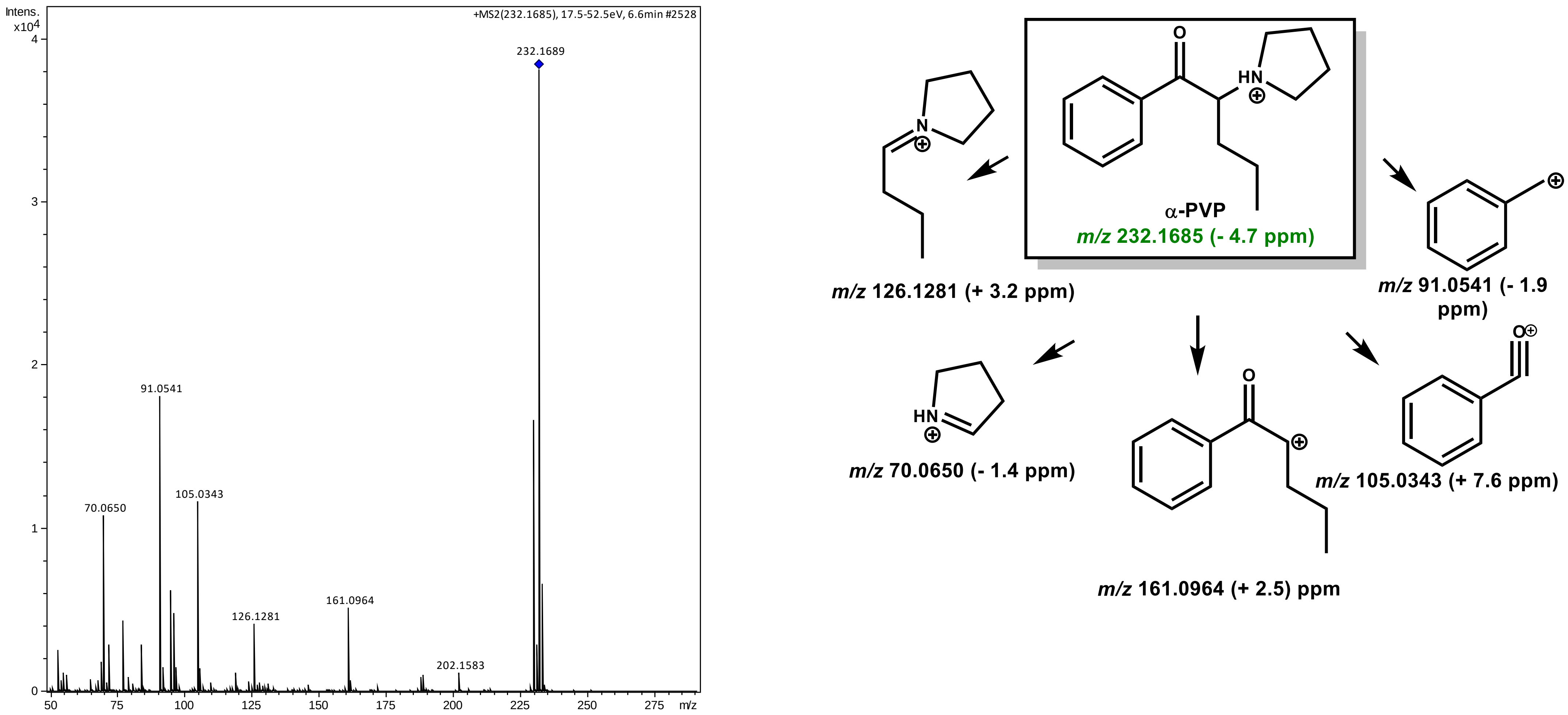

Figure S1. Tandem mass spectrum obtained for α-PVP by LC-HRMS (ESI+) and proposed structures for the diagnostic product ions.

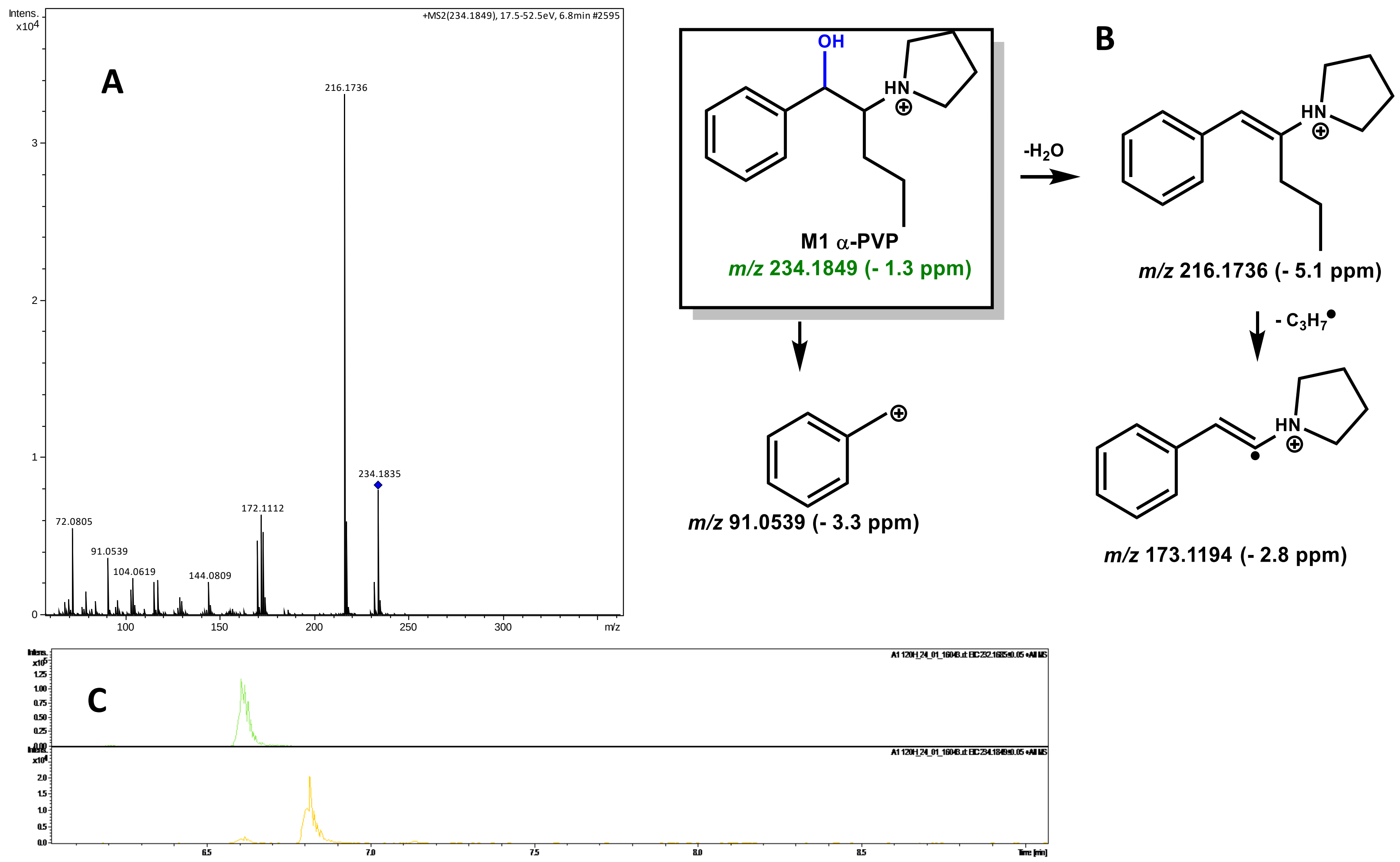

Figure S2. A. Tandem mass spectrum obtained for the Phase I metabolite **M1 α-PVP** by LC-HRMS (ESI+); **B**. Proposed structures for diagnostic product ions; and **C.** Extracted ion chromatogram of the parent cathinone and of **M1 α-PVP ions.**

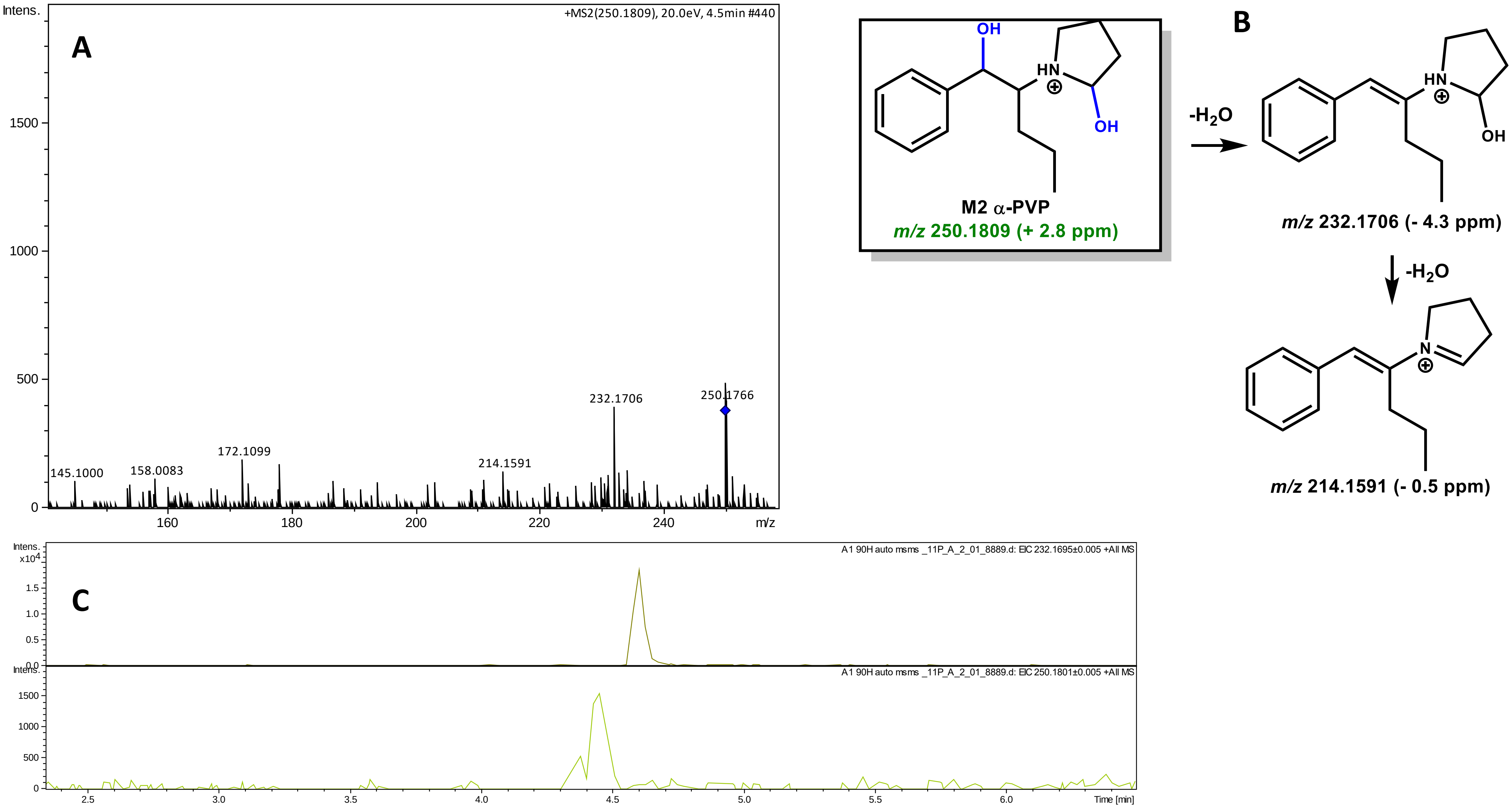

Figure S3. A. Tandem mass spectrum obtained for the Phase I metabolite **M2 α-PVP** by LC-HRMS (ESI+); **B**. Proposed structures for the diagnostic product ions; and **C.** Extracted ion chromatogram of the parent cathinone and of **M2 α-PVP ions.**

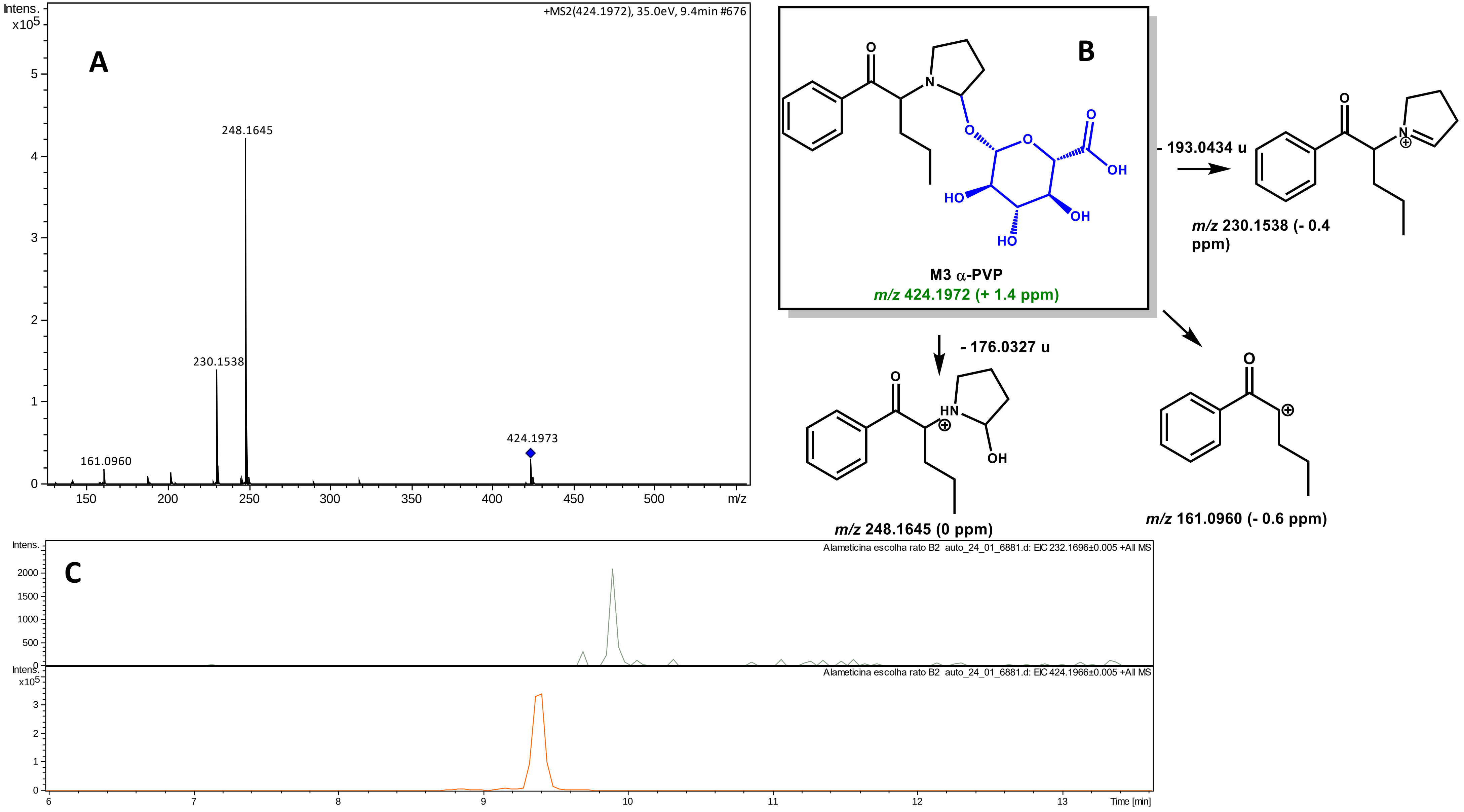

Figure S4. A. Tandem mass spectrum obtained for the Phase II metabolite **M3 α-PVP** by LC-HRMS (ESI+); **B.** Proposed structures for the diagnostic product ions; and **C.** Extracted ion chromatogram of the parent cathinone and of **M3 α-PVP ions.**

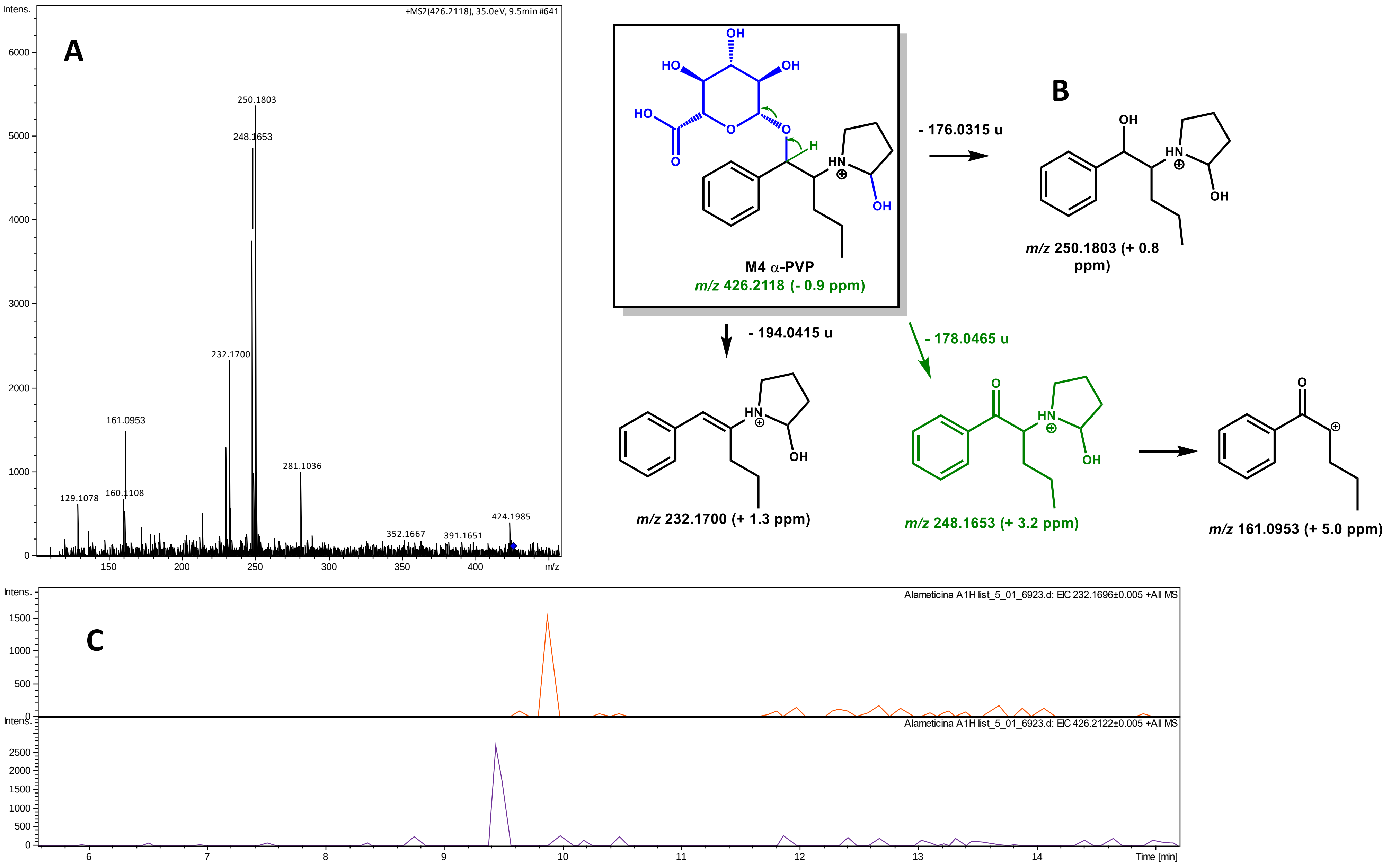

Figure S5. A. Tandem mass spectrum obtained for the Phase II metabolite **M4 α-PVP** by LC-HRMS (ESI+); **B.** Proposed structures and fragmentation pathways for the diagnostic product ions; and **C.** Extracted ion chromatogram of the parent cathinone and of **M4 α-PVP ions.**

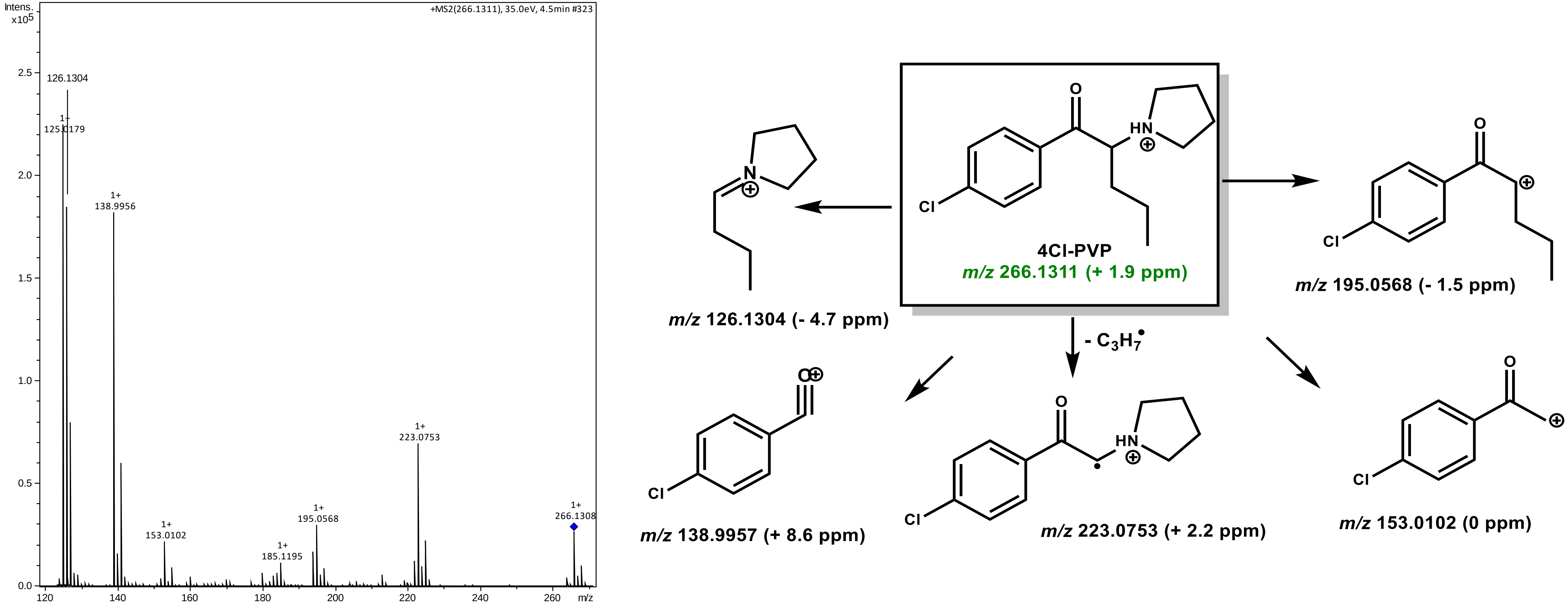

Figure S6. Tandem mass spectrum obtained for **4Cl-PVP** by LC-HRMS (ESI+) and proposed structures for the diagnostic product ions.

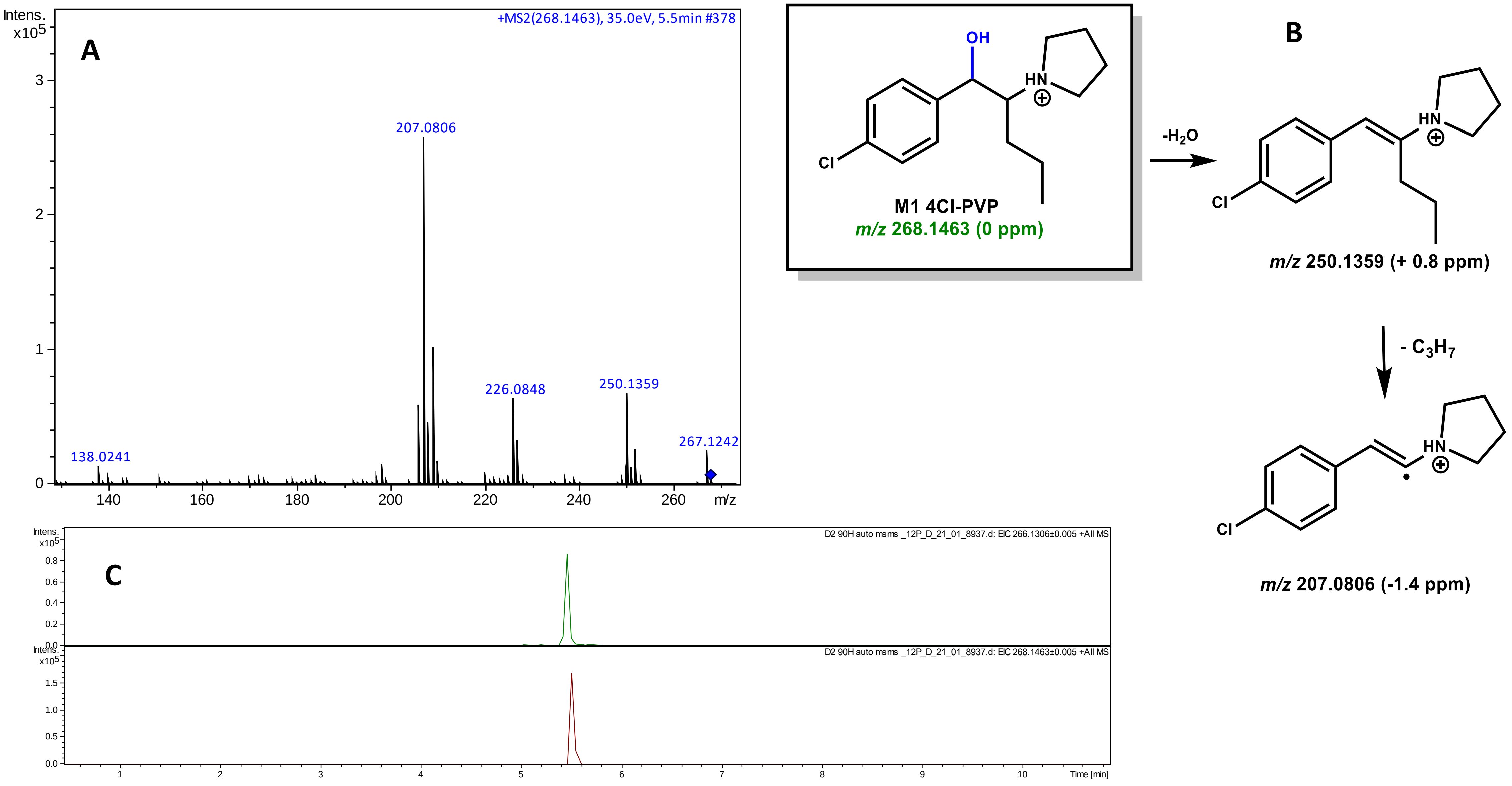

Figure S7. A. Tandem mass spectrum obtained for the Phase I metabolite **M1 4Cl-PVP** by LC-HRMS (ESI+); **B.** Proposed structures for the diagnostic product ions; and **C.** Extracted ion chromatogram of the parent cathinone and of **M1 4Cl-PVP** ions.

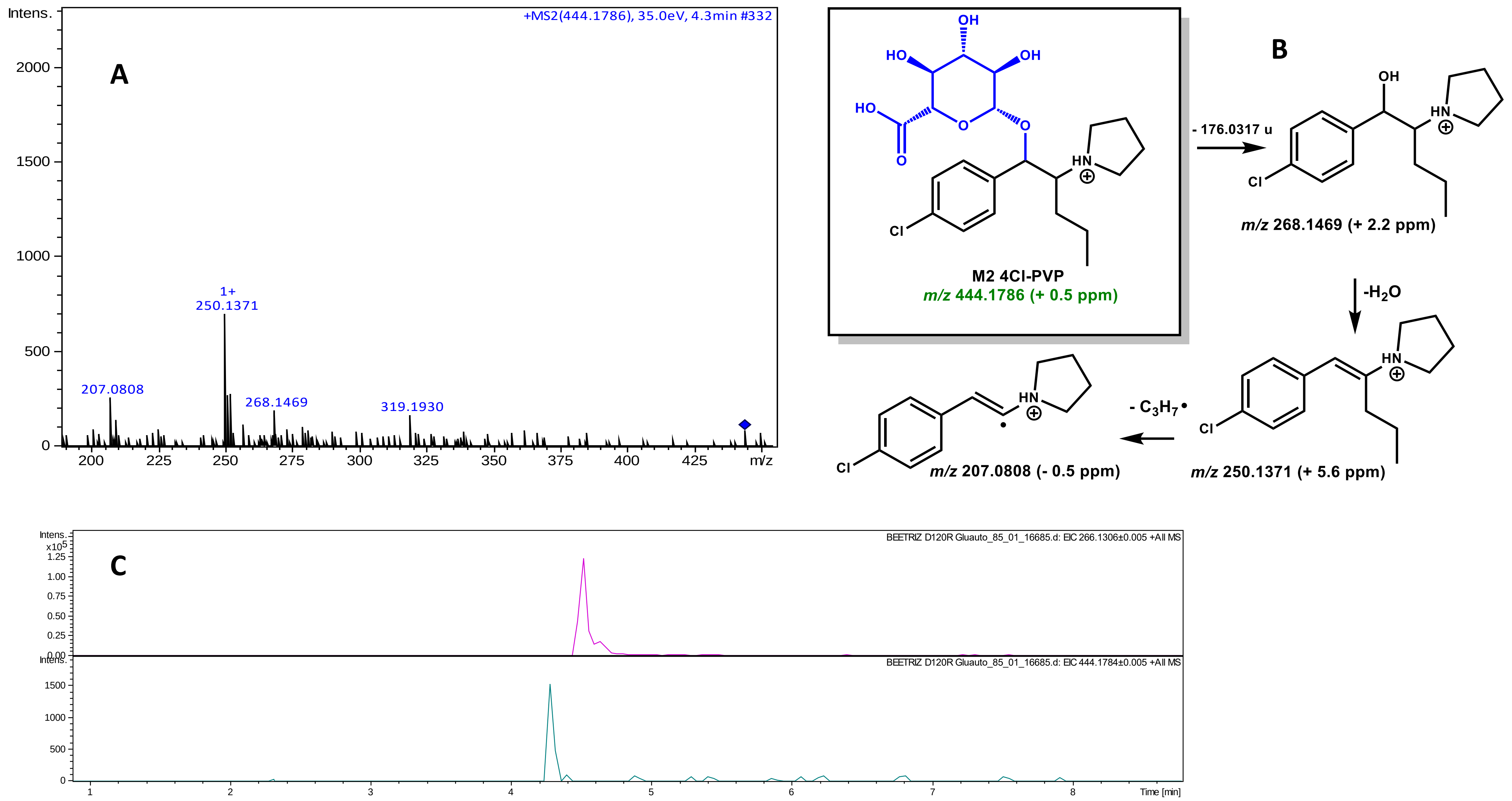

Figure S8. A. Tandem mass spectrum obtained for the Phase II metabolite **M2 4Cl-PVP** by LC-HRMS (ESI+); **B.** Proposed structures for the diagnostic product ions; and **C.** Extracted ion chromatogram of the parent cathinone and of **M2 4Cl-PVP** ions.

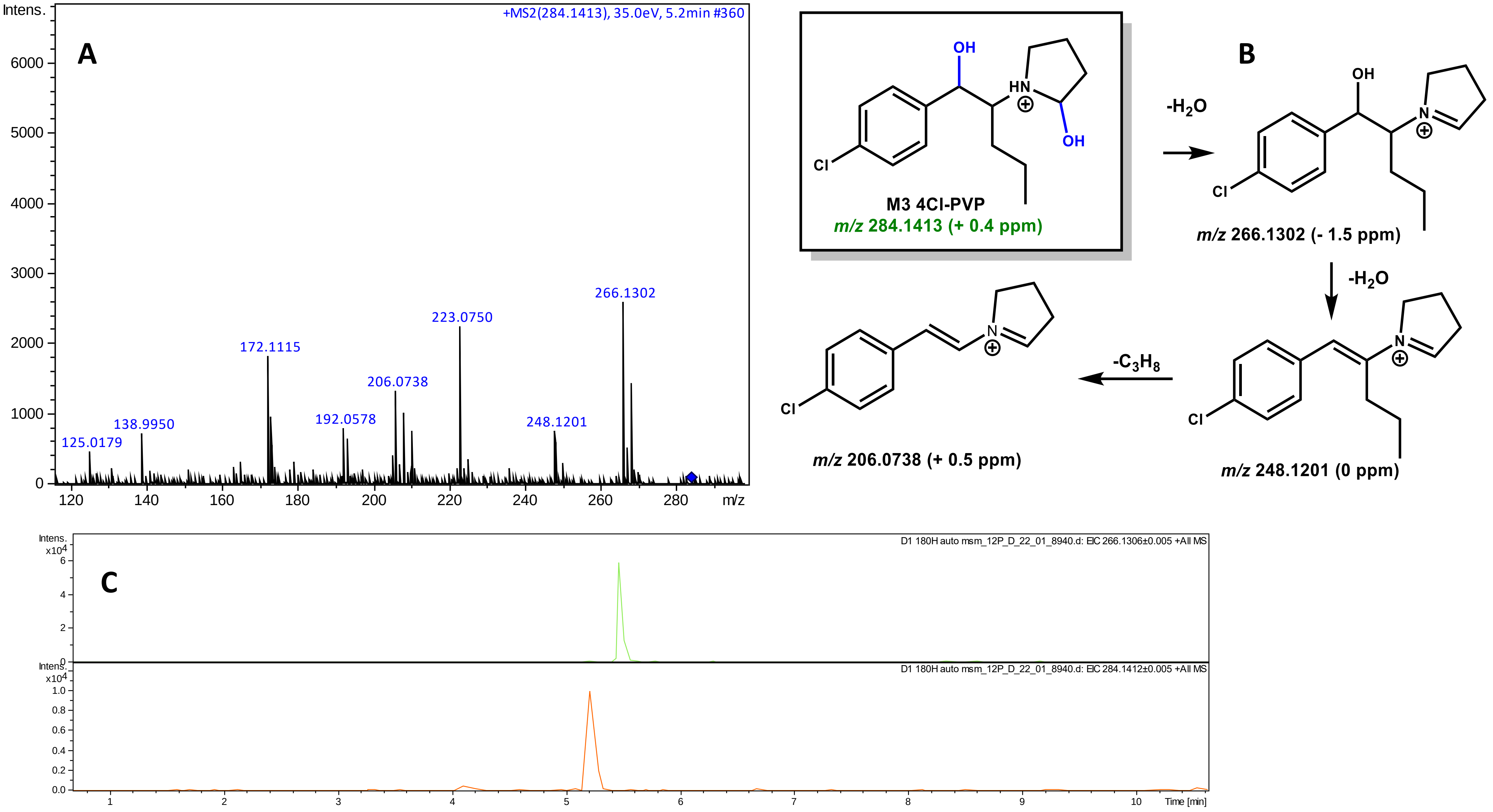

Figure S9. A. Tandem mass spectrum obtained for the Phase I metabolite **M3 4Cl-PVP** by LC-HRMS (ESI+); **B.** Proposed structures for the diagnostic product ions; and **C.** Extracted ion chromatogram of the parent cathinone and of **M3 4Cl-PVP** ions.

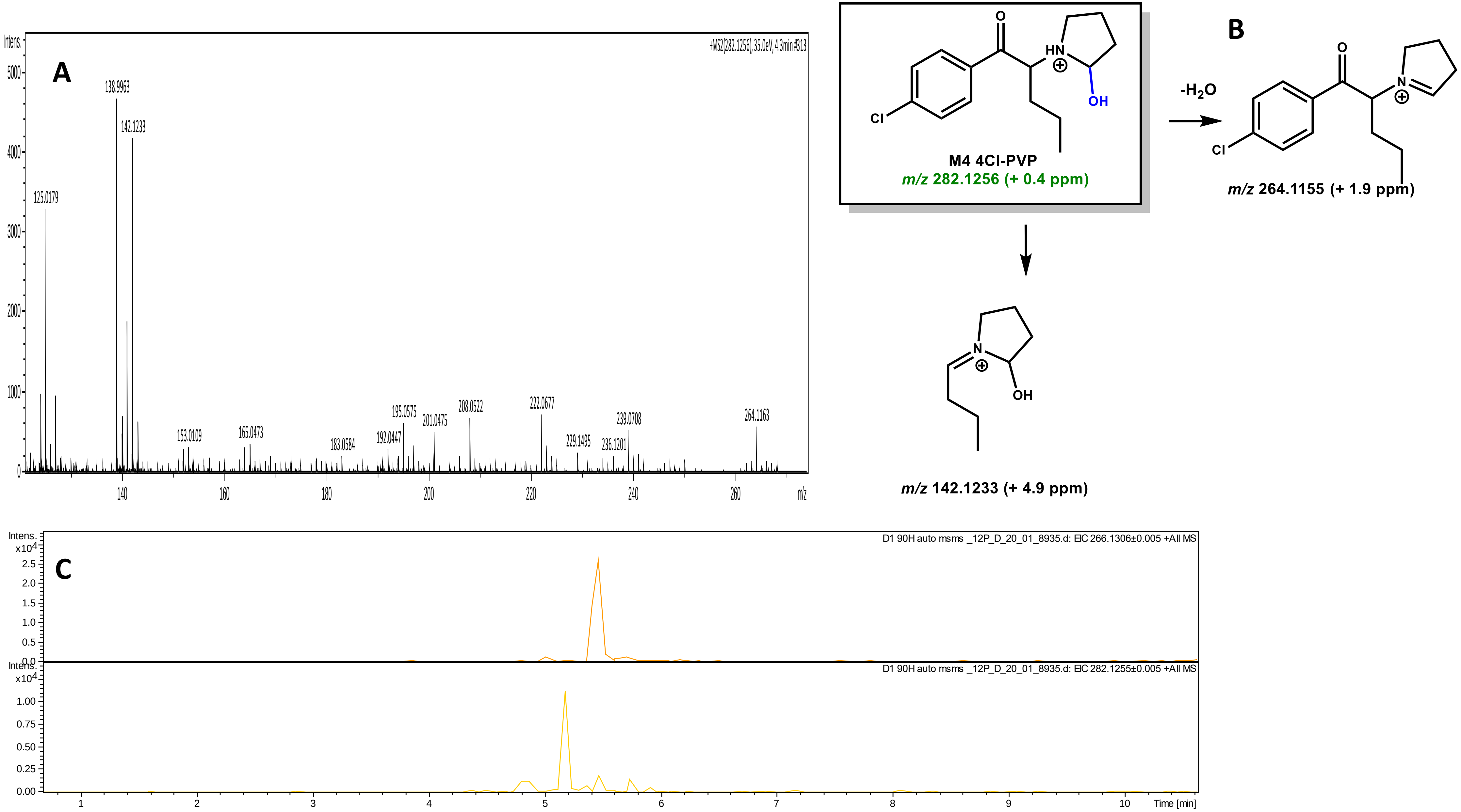

Figure S10. A. Tandem mass spectrum obtained for the Phase I metabolite **M4 4Cl-PVP** obtained by LC-HRMS (ESI+); **B.** Proposed structures for the diagnostic product ions; and **C.** Extracted ion chromatogram of the parent cathinone and of **M4 4Cl-PVP** ions.

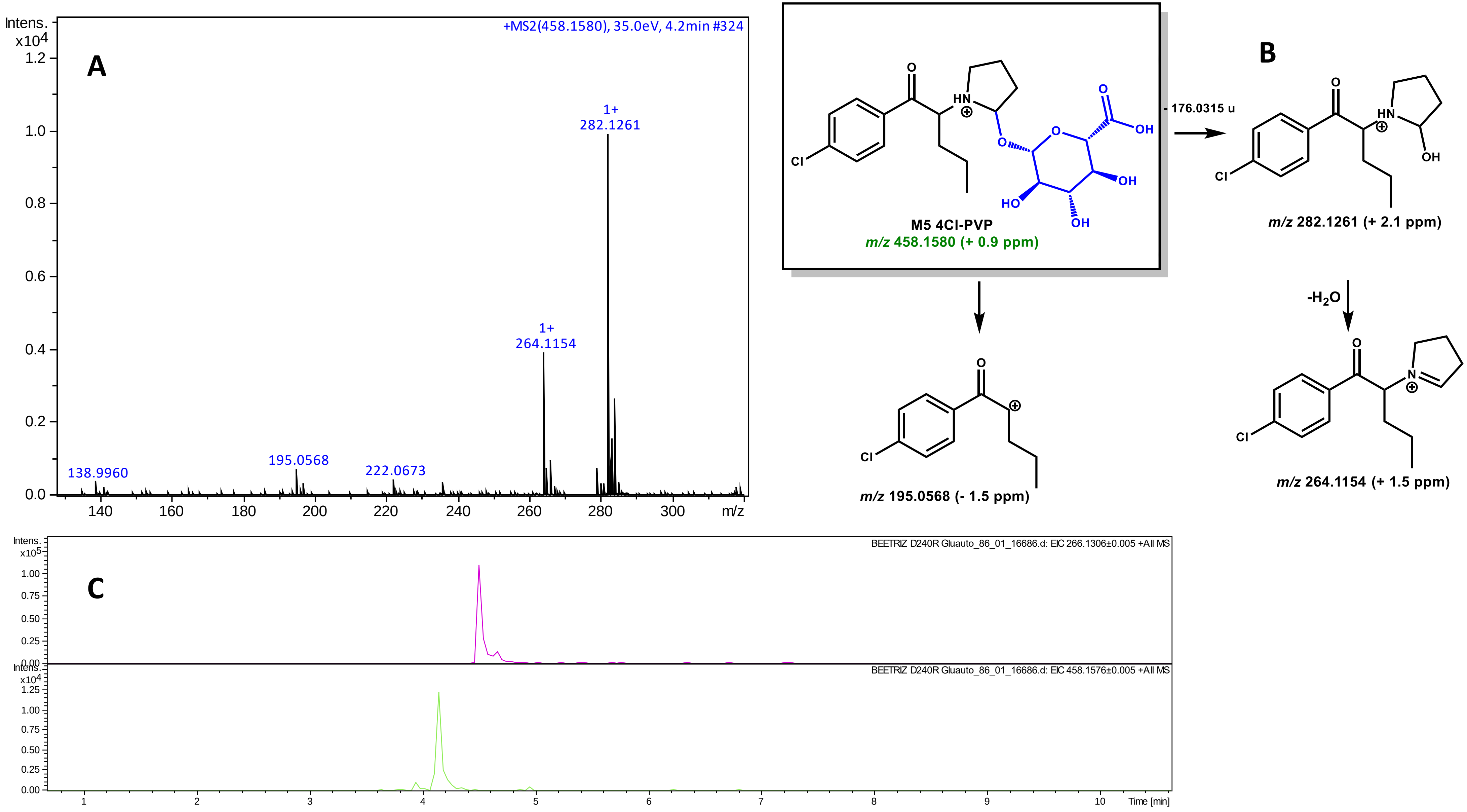

Figure S11. A. Tandem mass spectrum obtained for the Phase II metabolite **M5 4Cl-PVP** by LC-HRMS (ESI+); **B.** Proposed structures for the diagnostic product ions; and **C.** Extracted ion chromatogram of the parent cathinone and of **M5 4Cl-PVP** ions.

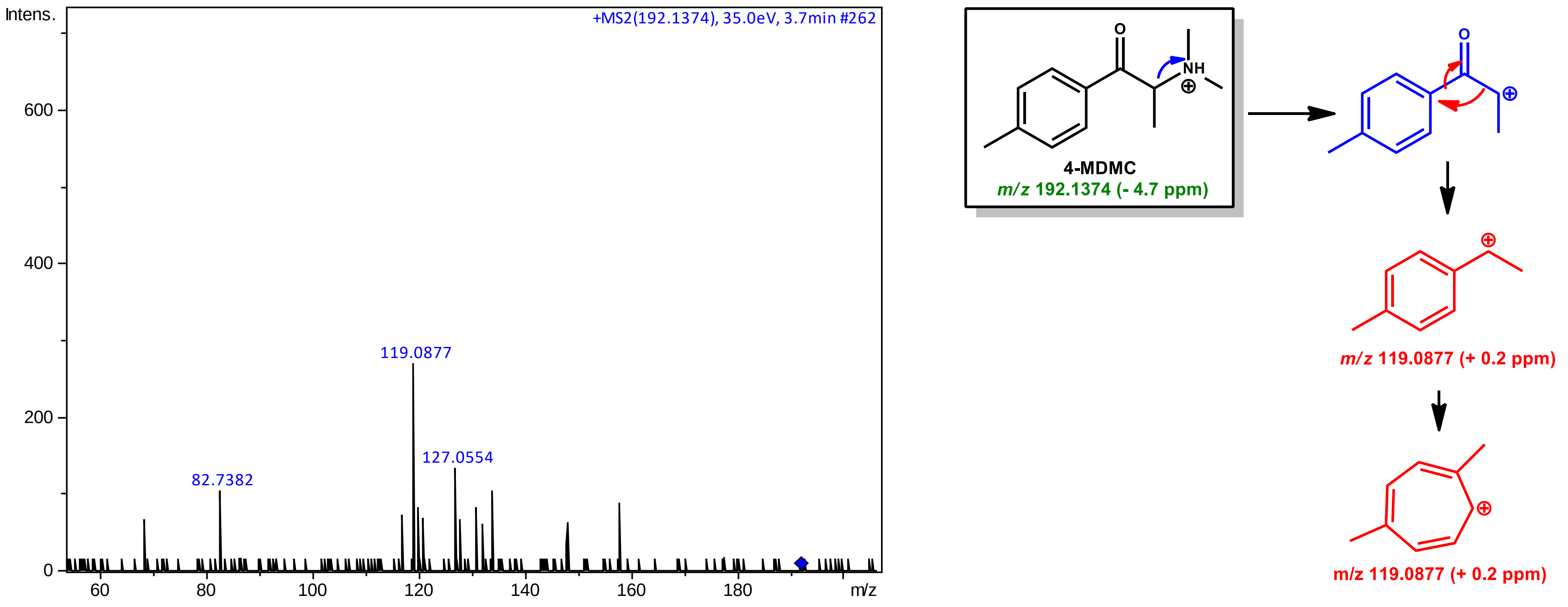

Figure S12. Tandem mass spectrum obtained for **4-MDMC** by LC-HRMS (ESI+) and proposed structure and fragmentation pathway for the diagnostic product ions identified.

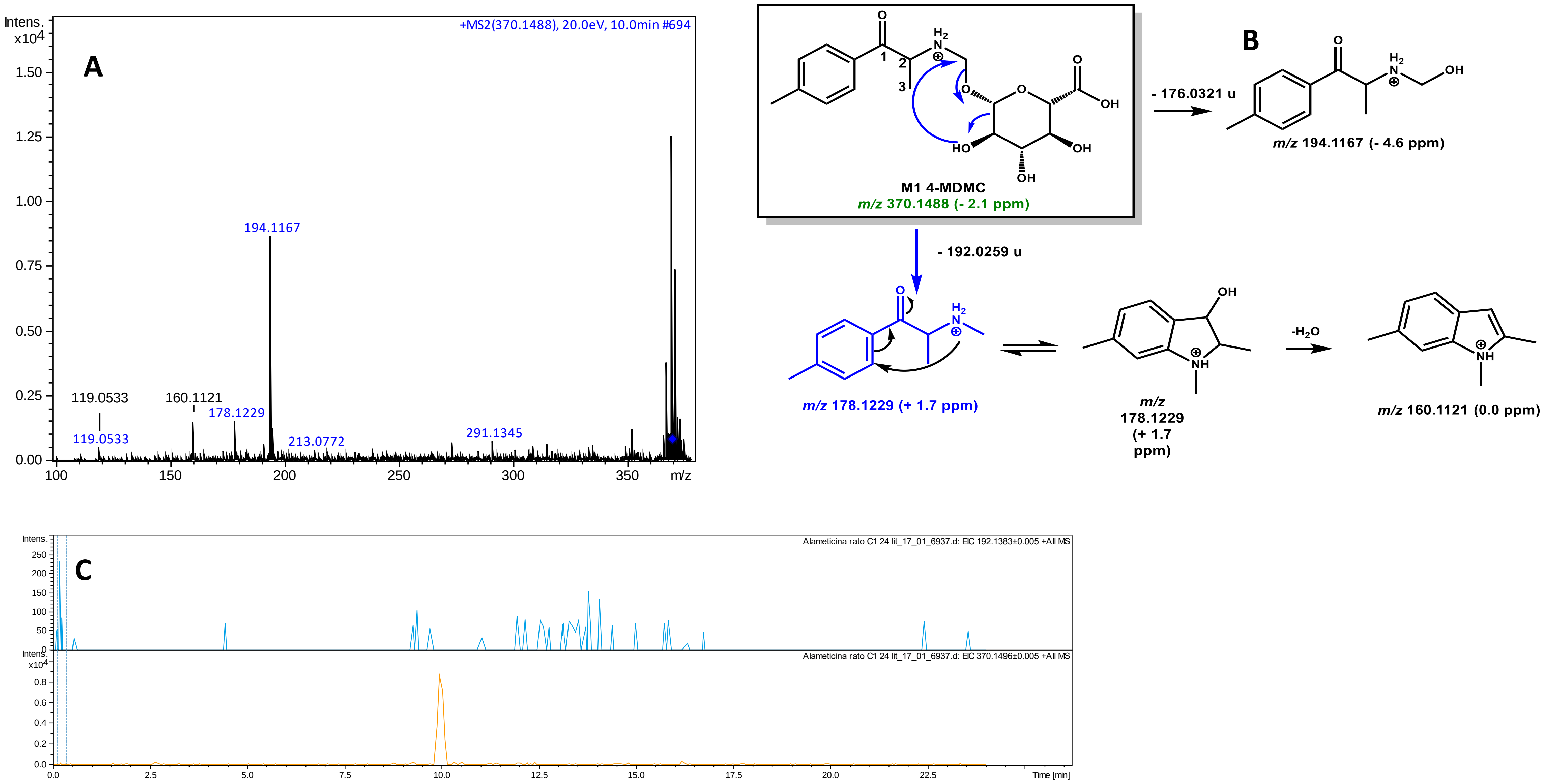

Figure S13. A. Tandem mass spectrum obtained for the Phase II metabolite **M1 4-MDMC** by LC-HRMS (ESI+); **B.** Proposed structures for the diagnostic product ions; and **C.** Extracted ion chromatogram of the parent cathinone and of **M1 4-MDMC** ions.

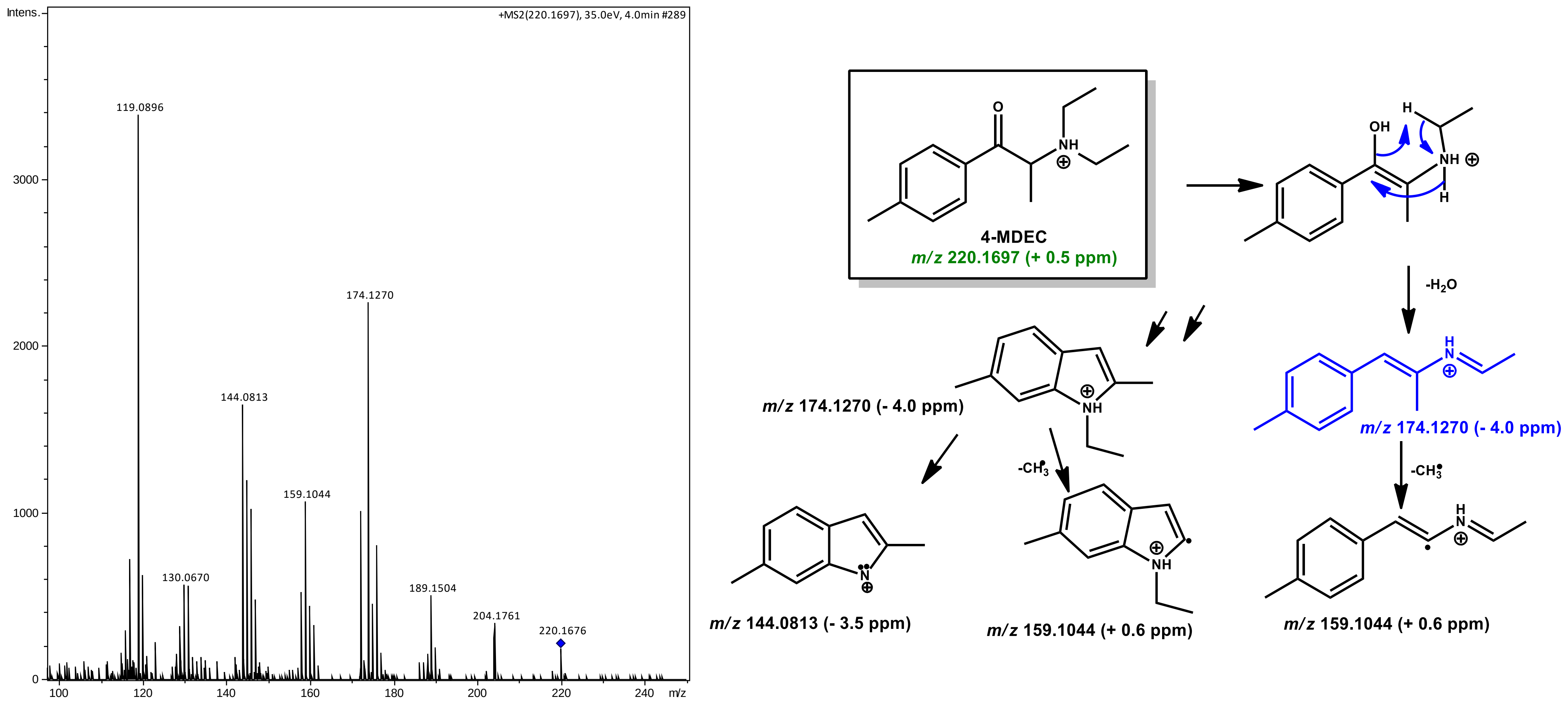

Figure S14. Tandem mass spectrum obtained for 4-MDEC by LC-HRMS (ESI+) and proposed structures for the diagnostic product ions identified.

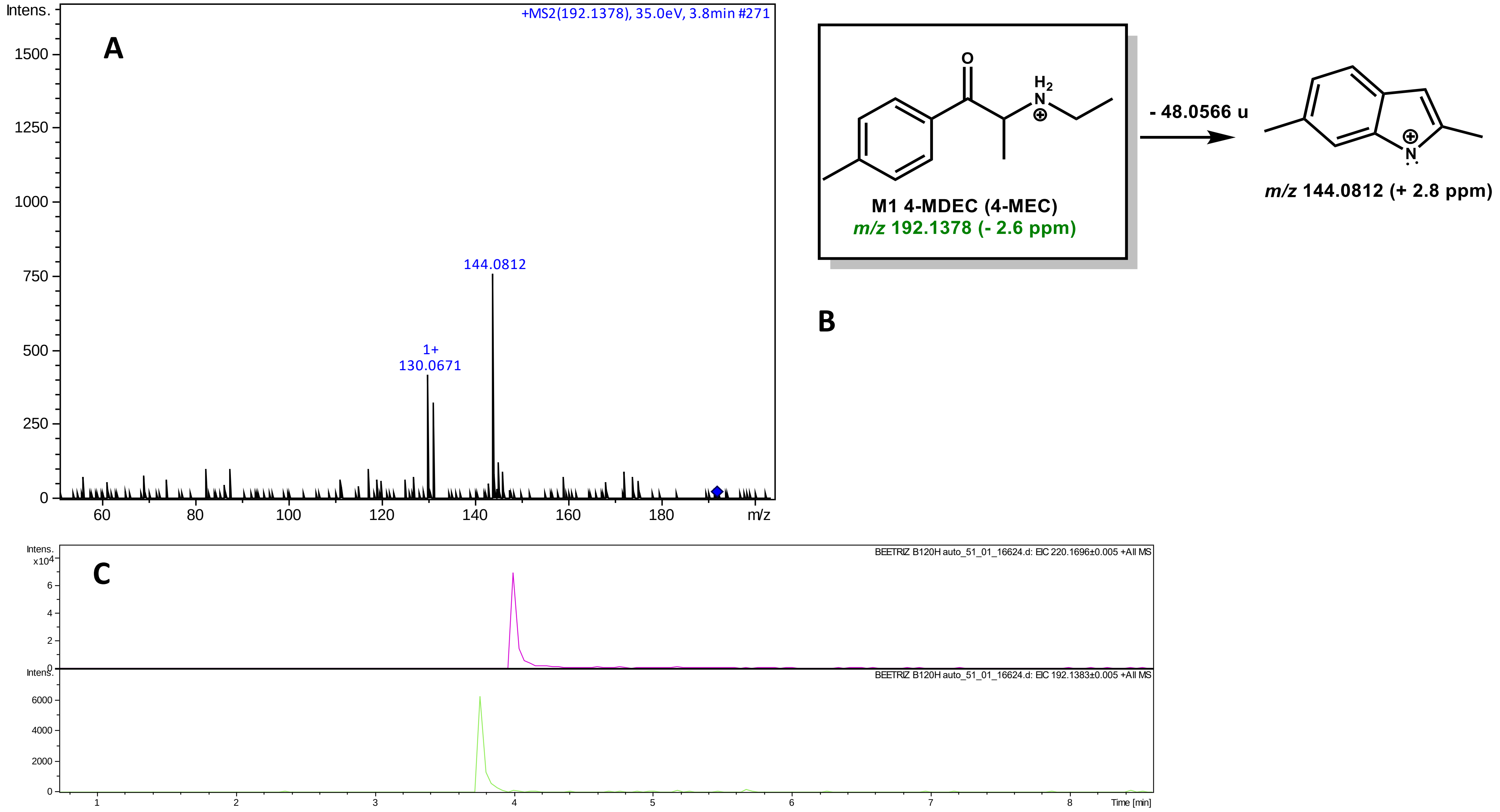

Figure S15. A. Tandem mass spectrum obtained for the Phase I metabolite **M1 4-MDEC** by LC-HRMS (ESI+); **B.** Proposed structures for the diagnostic product ions; and **C.** Extracted ion chromatogram of the parent cathinone and of **M1 4-MDEC** ions.

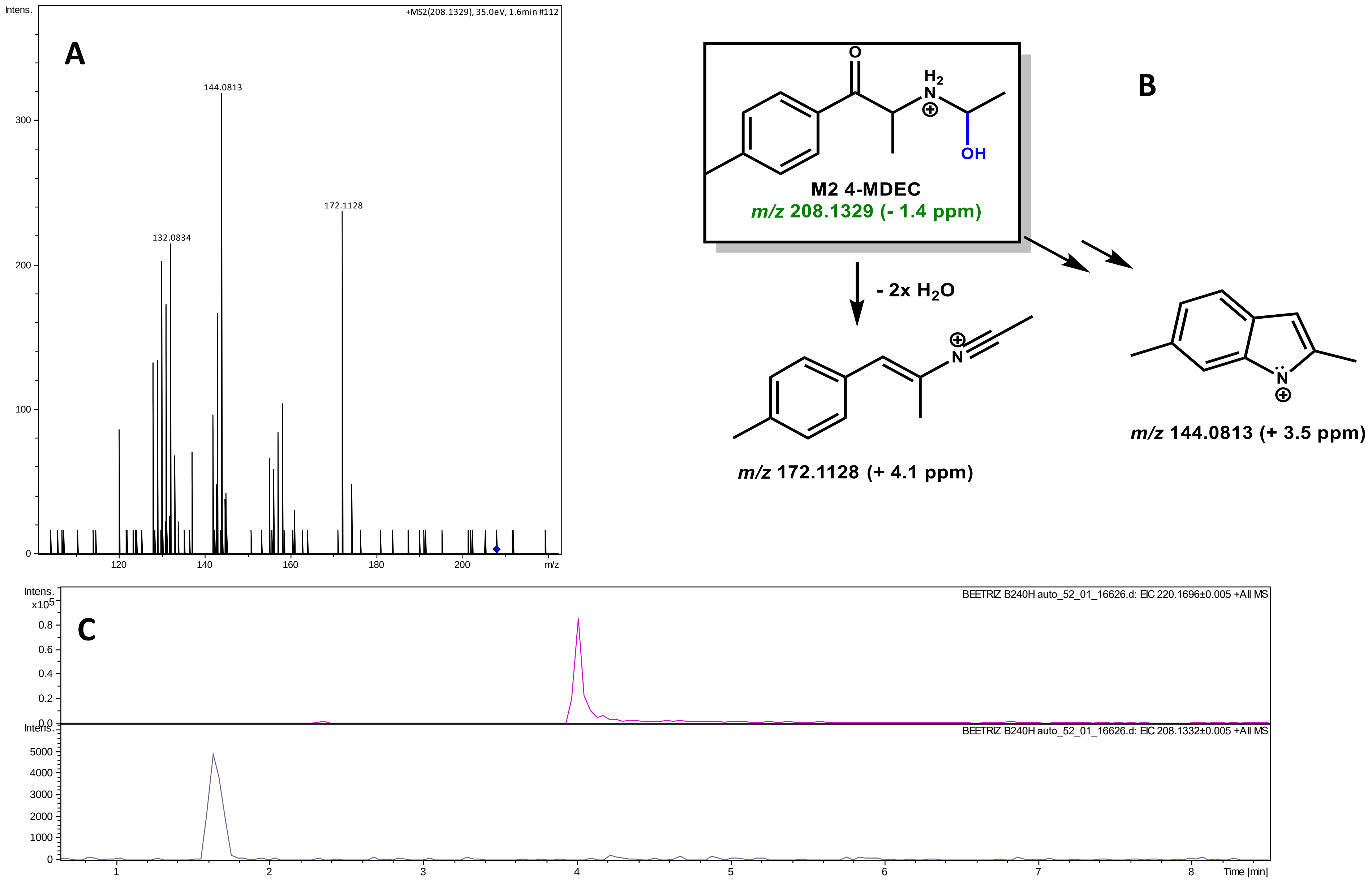

Figure S16. Tandem mass spectrum obtained for the Pase I metabolite **M2 4-MDEC** by LC-HRMS (ESI+); **B.** Proposed structures for the diagnostic product ions; and **C.** Extracted ion chromatogram of the parent cathinone and of **M2 4-MDEC** ions.

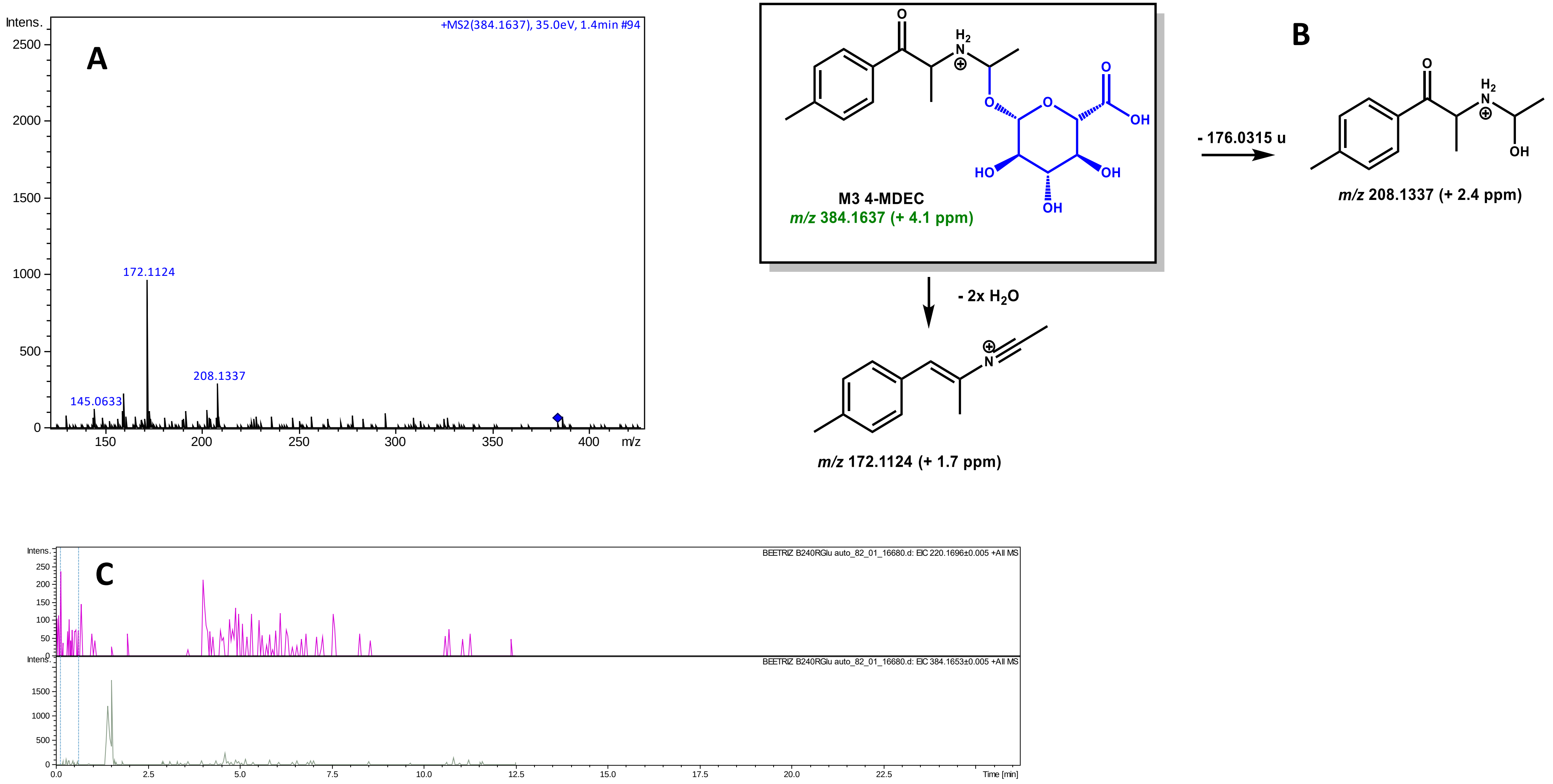

Figure S17. Tandem mass spectrum obtained for the Pase II metabolite **M3 4-MDEC** by LC-HRMS (ESI+); **B.** Proposed structures for the diagnostic product ions; and **C.** Extracted ion chromatogram of the parent cathinone and of **M3 4-MDEC** ions.

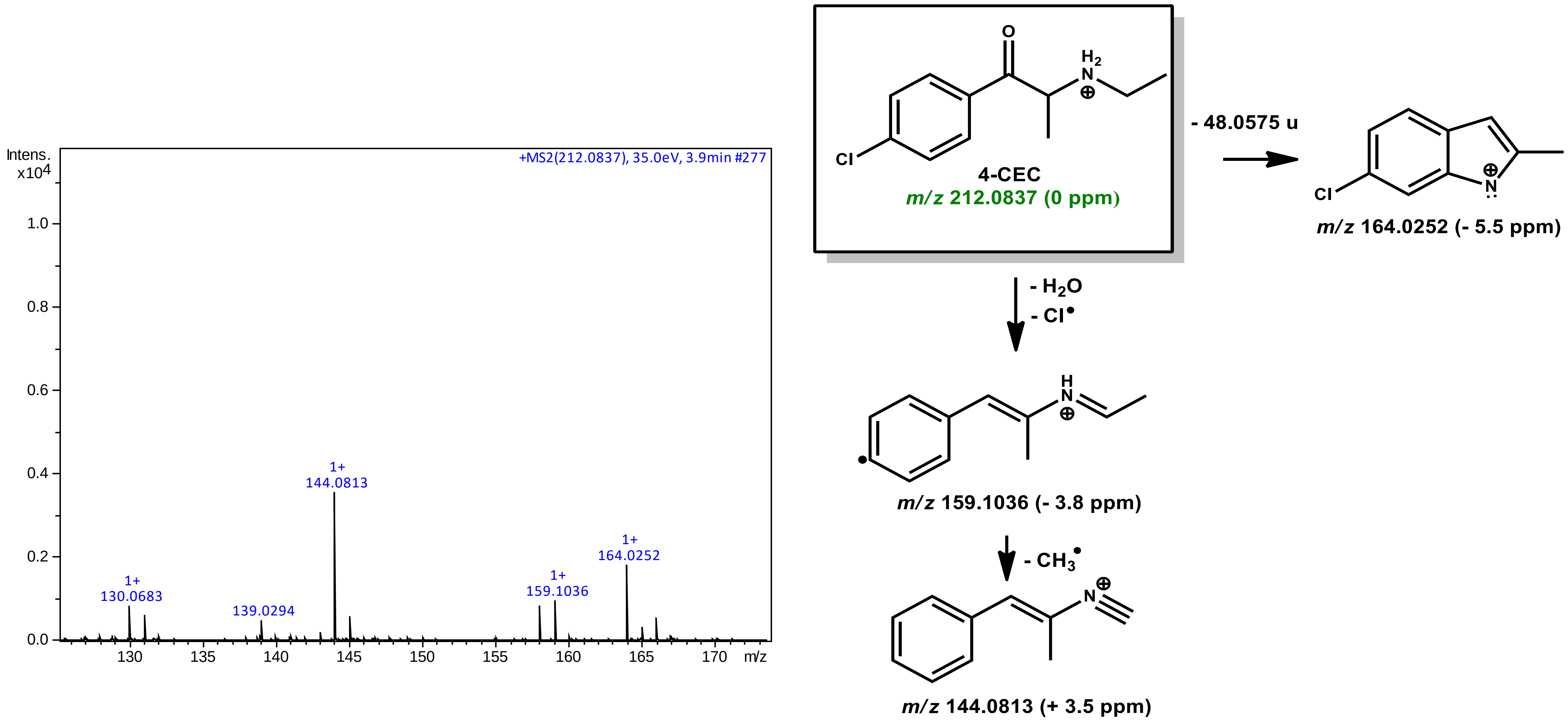

Figure S18. Tandem mass spectrum obtained for **4-CEC** by LC-QTOF-HRMS (ESI+) and proposed structures for the diagnostic product ions identified.

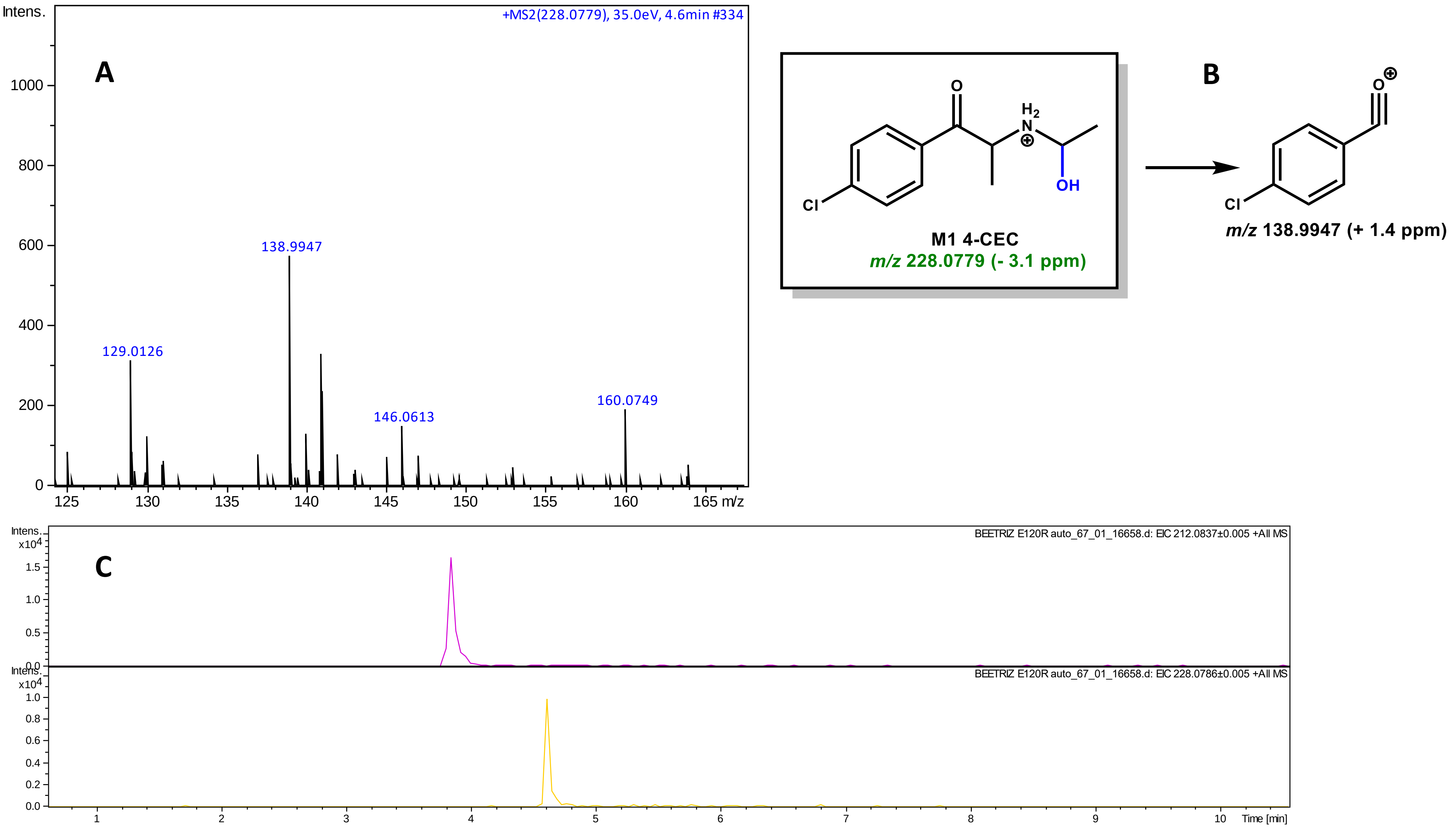

Figure S19. Tandem mass spectrum obtained for the Phase I metabolite **M1 4-CEC** by LC-HRMS (ESI+); **B.** Proposed structures for the diagnostic product ions; and **C.** Extracted ion chromatogram of the parent cathinone and of **M1 4-CEC** ions.

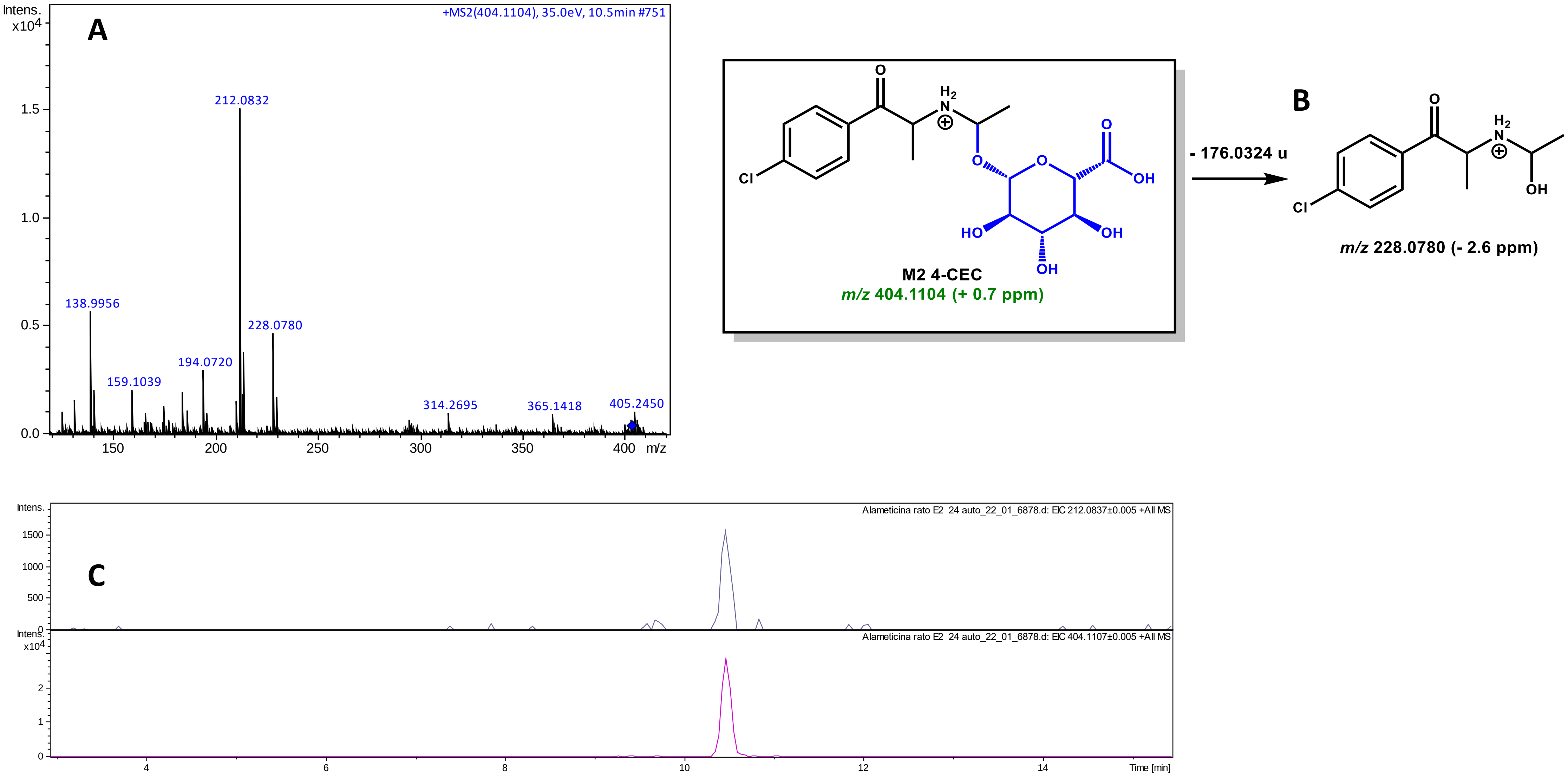

Figure S20. Tandem mass spectrum obtained for the Phase I metabolite **M2 4-CEC** by LC-HRMS (ESI+); **B.** Proposed structures for the diagnostic product ions; and **C.** Extracted ion chromatogram of the parent cathinone and of **M2 4-CEC** ions.
